# FOX transcription factors are common regulators of Wnt/β-catenin–dependent gene transcription

**DOI:** 10.1016/j.jbc.2023.104667

**Published:** 2023-04-01

**Authors:** Lavanya Moparthi, Stefan Koch

**Affiliations:** 1 Wallenberg Centre for Molecular Medicine (WCMM), Linköping University, Linköping, Sweden; 2 Department of Biomedical and Clinical Sciences (BKV), Linköping University, Linköping, Sweden

**Keywords:** cell signaling, transcription factor, Wnt pathway, proteomics, gene regulation

## Abstract

The Wnt/β-catenin pathway is a critical regulator of development and stem cell maintenance. Mounting evidence suggests that the outcome of Wnt signaling is determined by the collaborative action of multiple transcription factors, including members of the highly conserved forkhead box (FOX) protein family. However, the contribution of FOX transcription factors to Wnt signaling has not been investigated in a systematic manner. Here, we performed complementary screens of all 44 human FOX proteins to identify new Wnt pathway regulators. By combining β-catenin reporter assays with Wnt pathway–focused qPCR arrays and proximity proteomics of selected candidates, we determine that most FOX proteins are involved in the regulation of Wnt pathway activity. As proof-of-principle, we additionally characterize class D and I FOX transcription factors as physiologically relevant regulators of Wnt/β-catenin signaling. We conclude that FOX proteins are common regulators of Wnt/β-catenin–dependent gene transcription that may control Wnt pathway activity in a tissue-specific manner.

The Wnt/β-catenin signaling pathway, also referred to as the canonical Wnt pathway, is a central homeostatic signaling cascade, whose defining feature is the stabilization of β-catenin induced by WNT family proteins, which results in the differential expression of Transcription factor 7/Lymphoid enhancer-binding factor (TCF/LEF) target genes (1). Wnt/β-catenin signaling is critical for stem cell maintenance and proliferation, and dysregulation of Wnt pathway activity contributes to major diseases such as cancers. The WNT-induced transcriptional response is mediated predominantly by TCF/LEF transcription factors (2, 3). However, mounting evidence suggests that the context-specific outcome of Wnt/β-catenin signaling is determined by the collaborative action of various other transcription factors that control the expression of TCF/LEF target genes (4, 5, 6).

Among these additional Wnt pathway regulators are forkhead box (FOX) transcription factors. FOX proteins comprise one of the largest transcription factor families, with 44 main family members in humans that are subdivided into 19 classes (A through S) based on sequence similarity (7, 8, 9). Several lines of evidence across species suggest that there is substantial functional redundancy between FOX proteins that may not be limited to just closely related family members (10, 11, 12, 13). In the context of Wnt/β-catenin signaling, numerous FOX proteins have been shown to regulate pathway activity by altering WNT gene expression, affecting the subcellular shuttling of β-catenin and changing the composition of the TCF/LEF-associated transcriptional complex, among others (8, 14, 15). Accordingly, the role of some FOX proteins in, for example, carcinogenesis or lifespan control has been attributed to their activity in the Wnt pathway (16, 17). However, owing to the large number and divergent expression pattern of FOX transcription factors, the extent of their redundancy in Wnt pathway regulation remains poorly understood.

Because both the Wnt/β-catenin pathway and FOX transcription factors are critical for mammalian physiology and pathobiology, it is of considerable interest to better understand their reciprocal regulation. We therefore assessed the impact of the entire FOX transcription factor family on Wnt/β-catenin signaling using complementary, uniform gain-of-function screens in model cell lines. Overexpression of FOXs resulted in pronounced changes in Wnt pathway activator and inhibitor expression, which was associated with differential β-catenin/TCF reporter activity and TCF/LEF target gene transcription. Based on these findings, we characterize class D and I FOXs as Wnt signaling activators and inhibitors, respectively. We conclude that the induction of various FOX transcription factors causes a comprehensive transcriptional rewiring of the canonical Wnt pathway that is partially independent of β-catenin and TCF/LEF. Our study identifies FOX proteins as common regulators of Wnt/β-catenin–dependent gene transcription and provides mechanistic clues for the apparent functional redundancy of some FOXs in the Wnt signaling pathway.

## Results

### FOX transcription factors regulate Wnt/β-catenin signaling

**Figure 1:**
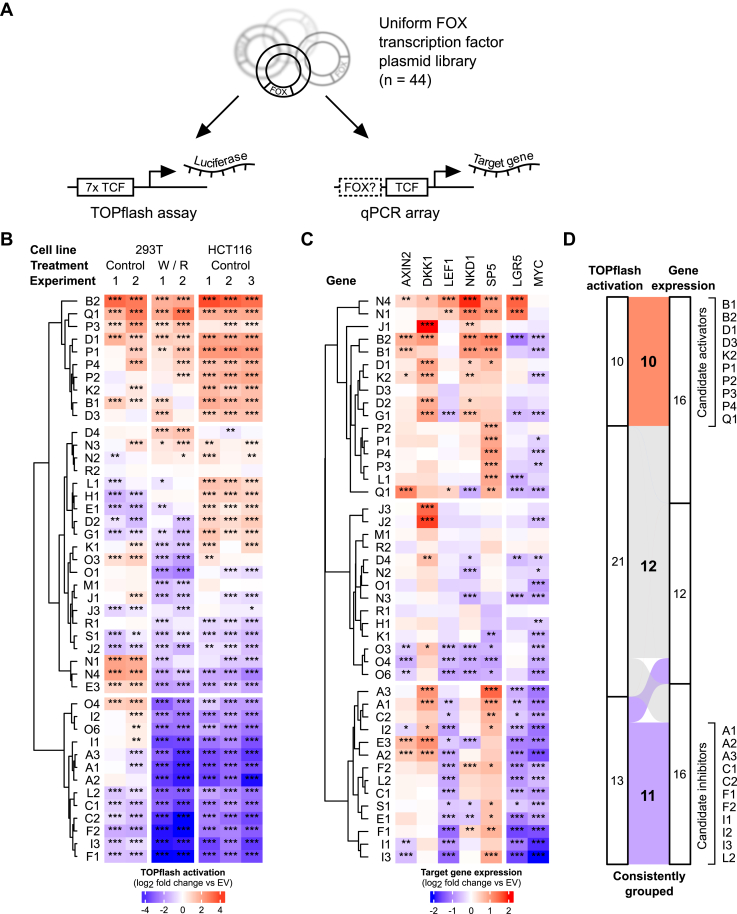
**FOX transcription factors are Wnt pathway regulators.***A*, schematic representation of the complementary gain-of-function screens used to identify Wnt pathway regulators in the FOX transcription factor family. *B*, heat map of β-catenin/TCF reporter (TOPflash) regulation by FOX transcription factors. Where indicated, cells were treated with Wnt3a and R-spondin 3 (W/R) conditioned media. *C*, heat map of TCF/LEF target gene expression changes following FOX gain-of-function. Gene expression was determined in 293T cells pretreated with 5 ng/ml recombinant human R-spondin 3. *D*, alluvial plot summarizing the grouping of FOX family members based on results from the combined screens. Data in (*B*) and (*C*) were normalized to the empty vector (EV) control in each column, with each cell showing the average of biological triplicates. Data for each experiment or target gene were analyzed using Dunnett’s post hoc test against EV following one-way ANOVA (∗∗∗*p* < 0.001, ∗∗*p* < 0.01, ∗*p* < 0.05). LEF, lymphoid enhancer-binding factor; TCF, transcription factor 7. See also Figs. S1–S3.

To identify potential new Wnt pathway regulators within the FOX family, we performed gain-of-function screens using two complementary standard assays for Wnt/β-catenin pathway activity: the β-catenin/TCF transcriptional reporter TOPflash (18) and a quantitative PCR (qPCR) array of multiple well-characterized TCF/LEF target genes (Fig. 1*A*). In both assays, we induced FOX proteins by transient overexpression of a functionally validated, uniform FOX plasmid library (19). TOPflash assays were performed in 293T cells with or without exogenous Wnt3a and R-spondin 3 (*i.e.*, Wnt pathway on or off), as well as in HCT116 colorectal cancer cells with constitutively active Wnt signaling. qPCR assays were done in 293T cells pretreated with R-spondin 3 to sensitize cells to subsequent pathway activation.

We observed that most FOX transcription factors significantly altered TOPflash activity across multiple independent experiments (Figs. 1*B*, and S1*A*). We performed unsupervised k-means clustering of the aggregated results to group FOXs into three discrete clusters (Fig. 1*B*). The first cluster contained several previously identified positive regulators of Wnt/β-catenin signaling, including FOXB2, FOXP1, FOXQ1, and FOXK2 (Table S1), as well as additional uncharacterized FOX proteins. Similarly, we identified a candidate inhibitor cluster that contained the well-studied negative Wnt pathway regulators FOXO4 and FOXF1/2, alongside others. For validation, we first repeated these experiments using the control plasmid FOPflash (18), which was negligibly regulated by most tested FOX proteins (Fig. S1*B*). Additionally, we performed TOPflash assays in 293T cells with genetic deletion of LRP5/6, β-catenin, or TCF/LEF/β-catenin (penta-KO) (20, 21), which are refractory to Wnt/β-catenin pathway activation (Fig. S1, *C* and *D*). In these cells, most FOX proteins had essentially no effect and did not synergize with Wnt3a/R-spondin 3 in TOPflash activation. Lastly, we repeated TOPflash experiments in HeLa (cervical cancer) and PC3 (prostate cancer) cell lines (Fig. S1*E*). Most putative Wnt pathway regulators differentially regulated TOPflash activity in these cells as well, especially in HeLa cells.

We then performed qPCR arrays of five *bona fide* TCF/LEF target genes in 293T cells (*AXIN2*, *DKK1*, *LEF1*, *NKD1*, *SP5*) (21), as well as two target genes that have been studied extensively in other contexts (*LGR5*, *MYC*) (Figs. 1*C* and S2). FOX transcription factors can control TCF/LEF target genes independently of β-catenin/TCF (20, 21), and *in silico* analyses of Wnt pathway–associated gene promoters highlighted numerous putative binding sites that could be occupied by FOX transcription factors alone or in combination with TCF/LEF family proteins (Fig. S3). Accordingly, we observed considerable variability in the regulation of different targets. Importantly, however, correlation analysis between the combined gene expression results and TOPflash data (Fig. 1, *B* and *C*) showed a significant interassay correlation (Pearson’s ρ = 0.21; *p* = 0.0001) that was primarily driven by the differential regulation of *AXIN2*, *LEF1*, and *MYC* (Fig. S2*C*). We thus clustered the gene expression data as before and observed that most FOX proteins were grouped consistently with the TOPflash data (Fig. 1, *C* and *D*). Collectively, this approach shortlisted ten likely activators and 11 candidate inhibitors of Wnt/β-catenin signaling in the FOX family (Fig. 1*D*), including FOXs that have not been studied in this context previously.

### FOX proteins control the expression of secreted Wnt pathway regulators

**Figure 2:**
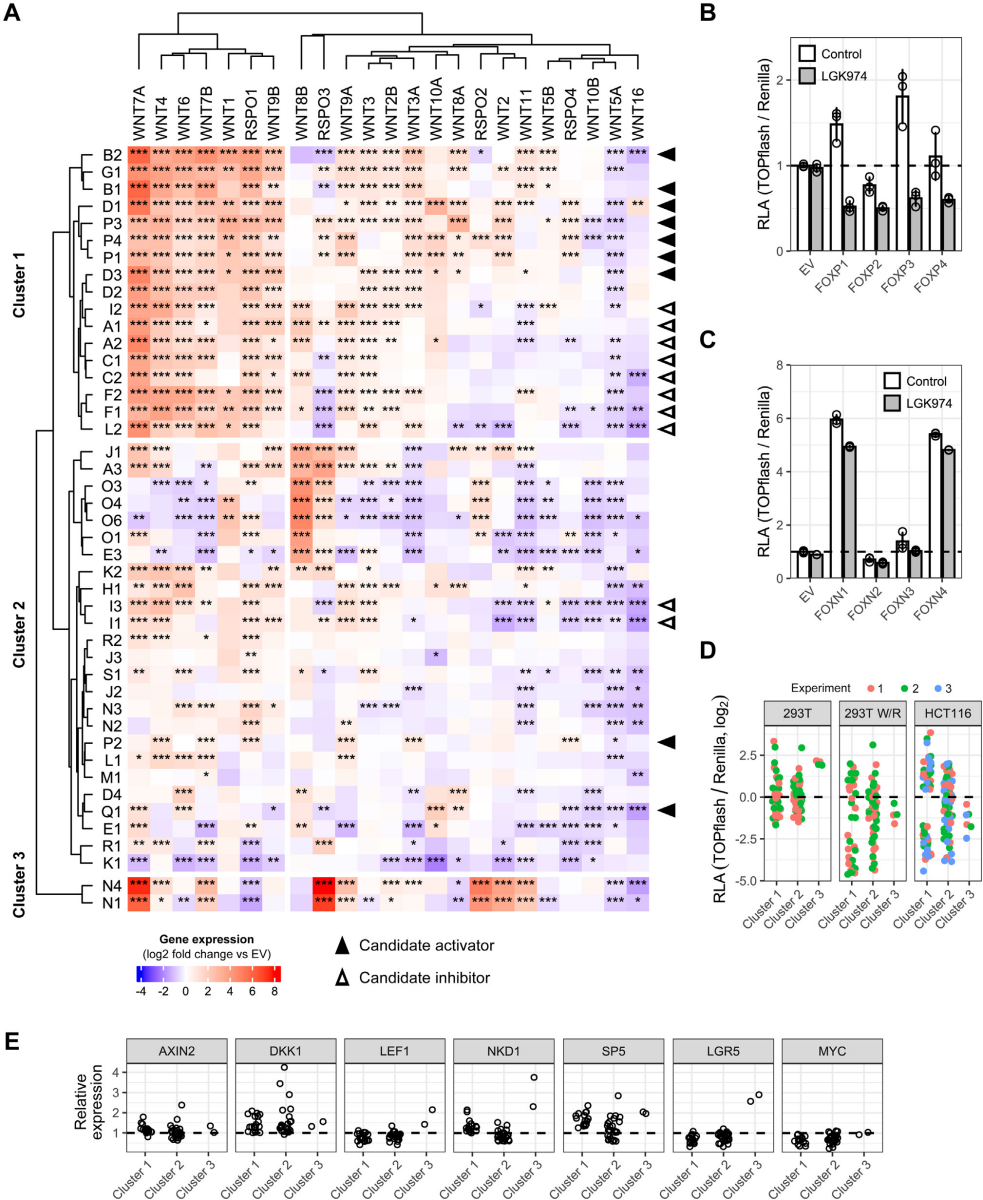
**FOX transcription factors regulate Wnt pathway agonist expression.***A*, heat map of WNT and R-spondin gene expression changes following FOX gain-of-function. Gene expression was determined in 293T cells pretreated with 5 ng/ml recombinant human R-spondin 3. Each cell represents the average of three biological replicates, normalized to empty vector (EV) control. *B* and *C*, TOPflash assay in 293T following FOXP (*B*) or FOXN (*C*) overexpression. Where indicated, cells were treated with 10 nM LGK974 to block WNT protein secretion. *D* and *E*, grouping of TOPflash (*D*) and qPCR results (*E*) from Figure 1 by clusters of Wnt pathway agonist expression. Data in panel (*A*) were analyzed using Dunnett’s post hoc test against EV following one-way ANOVA (∗∗∗*p* < 0.001, ∗∗*p* < 0.01, ∗*p* < 0.05). RLA, relative luciferase activity. See also Fig. S4.

We next explored different mechanisms by which FOX transcription factors may regulate Wnt/β-catenin signaling, starting with the induction of secreted agonists and inhibitors of the Wnt pathway (15). We assessed the expression of all 19 WNT genes, four R-spondins, and nine secreted Wnt pathway antagonists following FOX induction by qPCR (Figs. 2*A* and S4). We observed that several FOXs induced a group of secreted agonists that included *WNT4*, *WNT7A/B*, and *RSPO1*, among others. Consistently, inhibition of WNT protein secretion using the porcupine inhibitor LGK974 (22) had a more pronounced effect on TOPflash activation by FOXP1/3/4 than FOXP2 and FOXNs, which did not induce WNT genes to the same extent (Fig. 2, *B* and *C*). However, we observed the same pattern of Wnt pathway agonist induction by candidate activators as well as inhibitors, and across all FOX transcription factors, Wnt pathway agonist induction was not generally indicative of TOPflash activation or TCF/LEF target gene expression (Fig. 2, *D* and *E*). Similarly, while several FOXs differentially regulated various secreted Wnt pathway antagonists (Fig. S4), we did not observe any pattern consistent with their activity in Wnt pathway assays. Finally, we observed that most FOXs downregulated the noncanonical WNT proteins *WNT5A* and *WNT11* (Fig. 2*A*), which may prime cells further towards activation of canonical Wnt signaling (23). We conclude that FOX transcription factors control the expression of Wnt signaling agonists and antagonists but that this mechanism is insufficient to explain the activity of most FOX family members in the Wnt/β-catenin pathway.

### FOX proteins share a common proximity interactome with TCF/LEF

**Figure 3:**
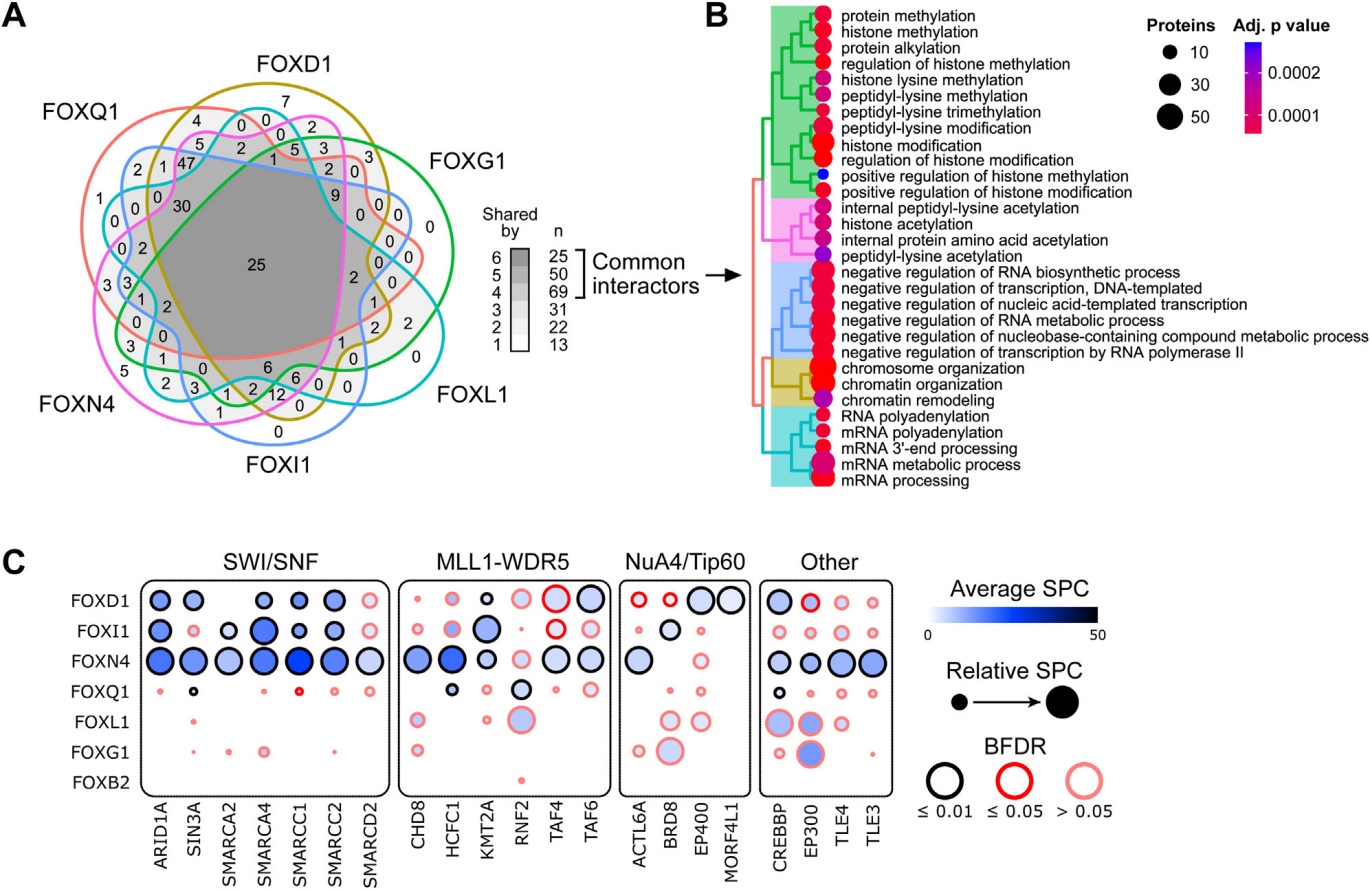
**Proximity proteomics identify common and unique FOX interactors.***A*, Venn diagram of high-confidence interactors of the indicated FOX proteins identified by TurboID-based proximity proteomics. *B*, Gene Ontology analysis of biological processes mediated by common FOX interactors. *C*, dot plot of important FOX interactors grouped by functional protein complex, indicated above. Proteomics results were from experiments with two to four biological replicates per bait or corresponding control condition. BFDR, Bayes false discovery rate; SPC, spectral count. See also Fig. S5.

FOX proteins occupy the promoter regions of various TCF/LEF target genes (21), and we have recently shown that FOXQ1 may control gene expression by recruiting similar transcription cofactors as TCF/LEF (20). To determine if this is also the case for other FOXs, we used TurboID-based proximity proteomics (24) to explore the interaction landscape of five additional FOXs, which displayed widely different transcriptional activity in Wnt pathway and forkhead box reporter assays (19). We identified 210 proteins as candidate interactors across all six tested FOXs, including our earlier TurboID-FOXQ1 data (Fig. 3*A*). Of these, 144 (68%) were associated with four or more FOX proteins, suggesting that they are common FOX interactors. Notably, this number is considerably higher than shared interactors identified in previous coimmunoprecipitation/mass spectrometry assays of 36 FOX transcription factors (25, 26) (Fig. S5*A*), likely due to the better performance of proximity proteomics in screens of chromatin-associated proteins (27). Gene Ontology analysis of these core interactors revealed a significant enrichment of proteins involved in RNA processing and histone modification, as expected (Fig. 3*B*).

We then asked to what extent the tested FOXs are associated with the same protein complexes as TCF/LEF. For this, we performed an enrichment analysis against a dataset of curated human protein complexes (28), including previously identified Tcf7l1, FOXQ1, and FOXB2 interactors (20, 29, 30). We observed that FOXQ1, FOXN4, FOXDI, and FOXI1, in particular, share a substantial number of interacting complexes with one another and Tcf7l1 (Figs. 3*C* and S5*B*). These included chromatin remodeling as well as histone acetylation and methylation complexes known to regulate TCF/LEF target gene expression (31). Of particular interest, we found that multiple FOXs associated with the histone methyltransferase KMT2A, which was recently identified as a critical regulator of TCF/LEF target gene expression (32). In contrast, we did not detect association of any FOX family member with β-catenin, TCF/LEF, or BCL9/L, that is, the core Wnt signaling–associated transcriptional complex.

### FOX D and I proteins are novel Wnt pathway regulators

**Figure 4:**
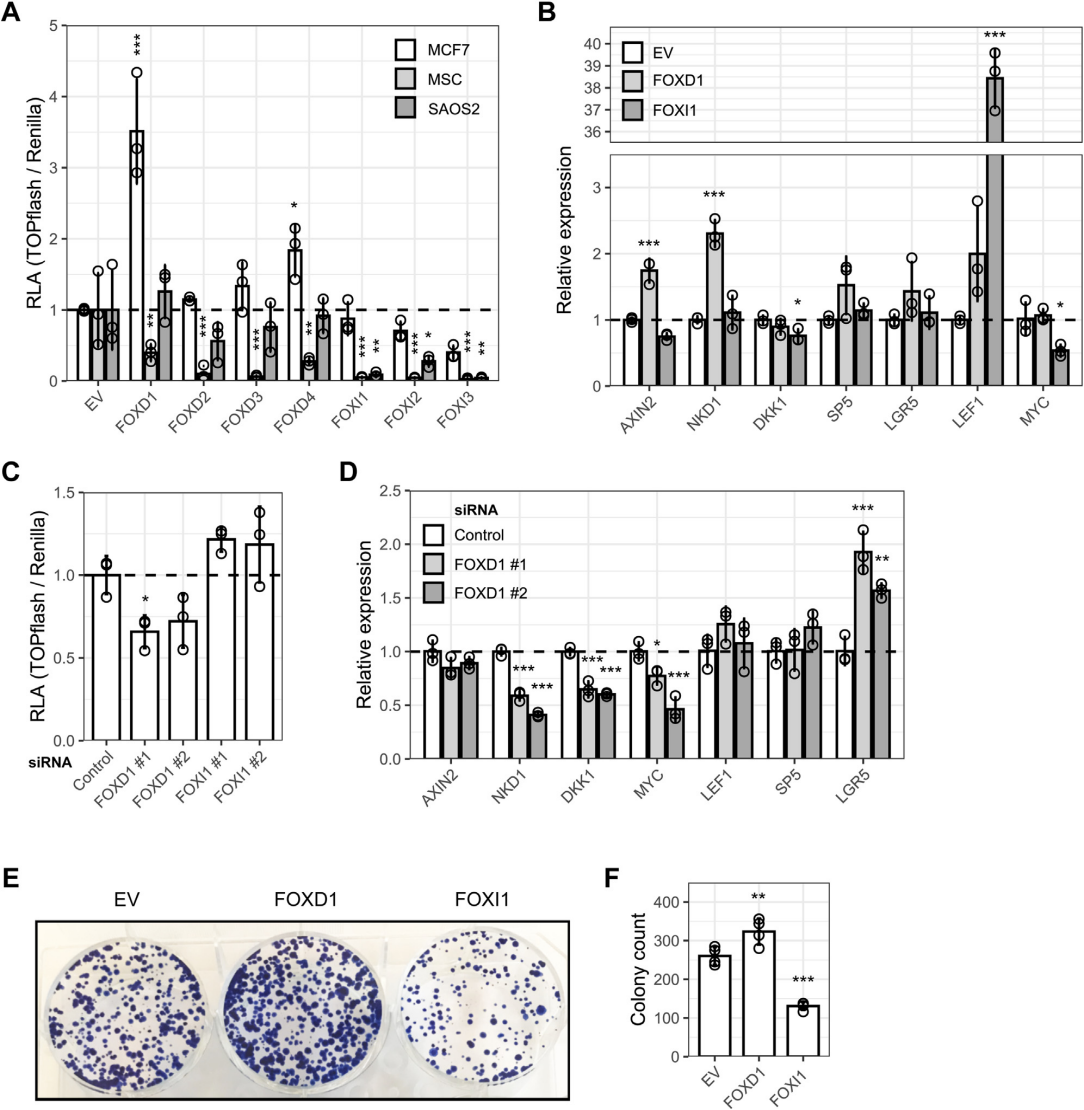
**FOXD and FOXI proteins are Wnt pathway regulators.***A*, TOPflash assay in MCF7, SAOS2, and mesenchymal stem cells (MSCs). MCF7 and MSC were pretreated with 5 μM GSK3 inhibitor CHIR99021. SAOS2 were pretreated with Wnt3a and R-spondin 3 conditioned media. *B*, TCF/LEF target gene expression in HCT116 following FOXD1 or FOXI1 expression, as determined by qPCR. *C*, TOPflash assay in 293T following FOXD1 or FOXI1 depletion with two independent siRNAs. *D*, TCF/LEF target gene expression in 293T following FOXD1 depletion, as determined by qPCR. *E*, representative image of colony formation assays in HCT116 following FOXD1 or FOXI1 gain-of-function. *F*, quantification of colony numbers from n = 4 biological replicates. Data in all panels were analyzed using Dunnett’s post hoc test against EV or siControl following one-way ANOVA (∗∗∗*p* < 0.001, ∗∗ *p* < 0.01, ∗*p* < 0.05). LEF, lymphoid enhancer-binding factor; RLA, relative luciferase activity; TCF, transcription factor 7. See also Fig. S6.

Our results so far suggested widespread control of Wnt/β-catenin signaling by FOX transcription factors. To validate some candidates of interest, we investigated FOXD and FOXI family members, which were recently highlighted as potential regulators of Wnt/β-catenin signaling (33, 34). FOXDs activated TOPflash in MCF7 breast cancer cells, but not in SAOS2 osteosarcoma cells or normal human mesenchymal stem cells (Fig. 4*A*), possibly indicating cell type–specific functions. In contrast, FOXIs inhibited Wnt reporter activity across all tested cell types. Moreover, overexpression of FOXD1 and FOXI1 in HCT116 and HeLa cells differentially regulated TCF/LEF target gene expression (Figs. 4*B* and S6*A*). Curiously, contrary to results from 293T, FOXI1 strongly induced LEF1 in these cells, possibly due to tissue-specific regulation of this gene (35).

Conversely, RNA interference of FOXD1 reduced TOPflash activity in 293T, while FOXI1 knockdown increased reporter activity (Fig. 4*C*). FOXD1 depletion also reduced the expression of multiple TCF/LEF target genes (Fig. 4*D*), although FOXI1 knockdown had no effect in these assays presumably due to low siRNA efficiency (Fig. S6, *B* and *C*). Finally, we determined the effect of FOXD1 and FOXI1 gain-of-function on colony formation in HCT116, which depend on β-catenin/TCF signaling for efficient proliferation (36). We observed that FOXD1 and FOXI1 significantly increased and decreased colony formation, respectively (Fig. 4, *E* and *F*). Taken together, these findings support a physiologically relevant role of class D and I FOXs in the regulation of Wnt/β-catenin signaling.

### FOXD1 and FOXI1 have discrete modes of action in the Wnt pathway

**Figure 5:**
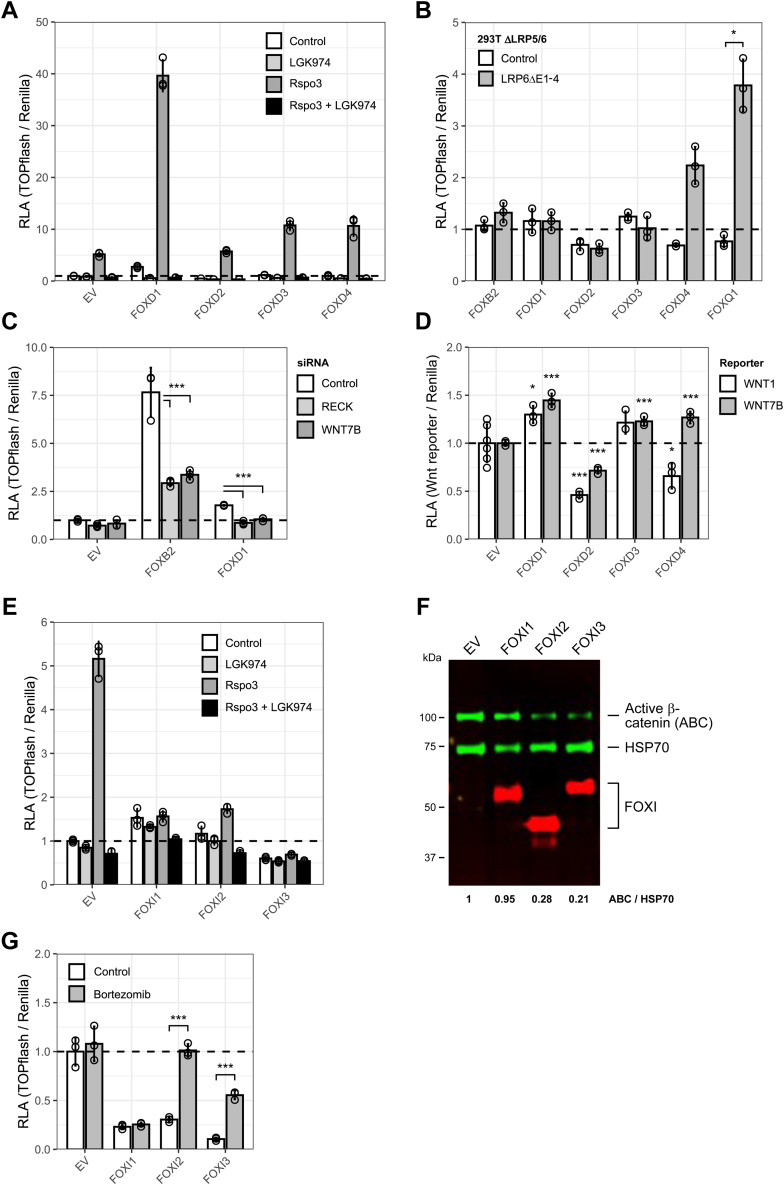
**FOXD1 and FOXI1 have discrete functions in the Wnt pathway.***A*, TOPflash assay in 293T. Where indicated, cells were treated with 5 ng/ml recombinant human R-spondin 3 (Rspo3) or 10 nM porcupine inhibitor LGK974. *B*, TOPflash assay in 293T ΔLRP5/6. Where indicated, cells were transfected with constitutively active LRP6 (LRP6ΔE1-4) to uncouple Wnt/β-catenin pathway activation from LRP5/6 engagement by WNT proteins. FOXQ1 and FOXB2 were included as positive and negative controls, respectively. Data were normalized to the corresponding empty vector control. *C*, TOPflash assay in 293T following *WNT7B* or *RECK* depletion. *D*, WNT1 and WNT7B promoter reporter assay in 293T. *E*, TOPflash assay in 293T. Where indicated, cells were treated with 5 ng/ml recombinant human R-spondin 3 (Rspo3) or 10 nM LGK974. *F*, immunoblot of nonphosphorylated, active β-catenin (ABC) levels in Wnt3a-treated 293T following *FOXI* expression. The relative ratio of ABC *versus* HSP70 housekeeping control is indicated below. *G*, TOPflash assay in HCT116. Where indicated, cells were treated with 100 nM proteasome inhibitor bortezomib. Data in (*B*) and (*G*) were analyzed using an unpaired Welch’s *t* test with Bonferroni-Hochberg correction for multiple testing. Data in (*C*) and (*D*) were analyzed using Dunnett’s post hoc test against EV or siControl following one-way ANOVA (∗∗∗*p* < 0.001, ∗*p* < 0.05). RLA, relative luciferase activity. See also Fig. S7.

Given that FOXD1-3 strongly induced the expression of multiple WNT genes, we asked if this mechanism may sufficiently explain their activity in the Wnt pathway, as we have previously shown for FOXB2 (29). Consistent with this hypothesis, TOPflash activation by FOXDs was blocked by treatment with the porcupine inhibitor LGK974 (Fig. 5*A*). Moreover, FOXD1-3 were unable to activate TOPflash in 293T ΔLRP5/6 cells expressing constitutively active LRP6 lacking its ligand-binding extracellular domain (Fig. 5*B*). Interestingly, FOXD4 still activated TOPflash in this assay, suggesting that its mode of action is distinct. Because *WNT7A/B* were among the most highly induced WNT genes, we depleted *WNT7B* or the WNT7 coreceptor *RECK* by RNA interference using previously validated siRNAs (29). Under these conditions, FOXD1 was unable to activate TOPflash in 293T (Fig. 5*C*). Additionally, FOXD1 and FOXD3 in particular were able to modestly activate WNT7B and WNT1 promoter reporters, consistent with direct transcriptional regulation of these genes (Fig. 5*D*). Taken together, these results suggest that FOXD1-3 activate Wnt signaling by inducing agonistic WNT proteins.

FOXIs, on the other hand, had a limited impact on WNT gene expression, and their inhibitory activity was not affected by treatment with the porcupine inhibitor LGK974 (Fig. 5*E*). Several FOX family members such as FOXOs inhibit Wnt signaling by competitive binding of β-catenin (37). We observed that FOXIs inhibited TOPflash activation following β-catenin stabilization or overexpression (Fig. S6, *D* and *E*). However, we did not detect physical interaction of FOXIs with β-catenin (Fig. S7*A*), arguing against β-catenin sequestration. Instead, we noted a destabilization of β-catenin protein by FOXIs and reduced nuclear β-catenin levels at least in the presence of FOXI2/3 (Figs. 5*F* and S7*B*). Consistently, proteasomal inhibition released the effect of FOXI2/3 on TOPflash activity in HCT116 (Fig. 5*G*). We conclude that FOXIs may inhibit Wnt signaling at least in part by promoting the degradation of β-catenin.

**Figure 6:**
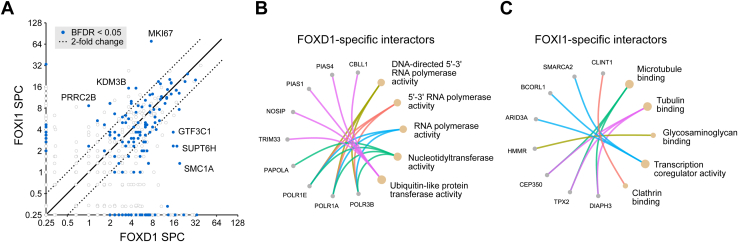
**FOXD1 and FOXI1 recruit distinct transcription cofactors.***A*, scatter plot of candidate interactors of FOXD1 and FOXI1 identified by TurboID proximity proteomics. Some interactors that are enriched in one of the samples are highlighted. Hits along the axes were considered specific interactors. *B* and *C*, functional grouping of specific interactors of (*B*) FOXD1 and (*C*) FOXI1, based on Gene Ontology molecular function terms. BFDR, Bayes false discovery rate; SPC, average spectral count. See also Figs. S8 and S9.

FOXD1 and FOXI1 have opposing functions in the Wnt pathway despite a largely overlapping interactome and virtually identical DNA recognition motifs (Fig. S8*A*). It is thus likely that their effects are mediated by few specific cofactors. Pairwise analysis of the TurboID data confirmed that most identified interactors were shared between FOXD1 and FOXI1 (Fig. 6*A*). Accordingly, pathway-focused analyses did not reveal any significant results due to the low number of unique hits. However, among the FOXD1-exclusive hits, we detected the ubiquitin/SUMO ligases PIAS4 and TRIM33 (Fig. 6*B*), which have been implicated in the regulation of Wnt signaling (38, 39, 40). We also noted that FOXD1 specifically interacted with JUNB (Fig. S8*B*), which has been shown to control the expression of multiple WNT genes including *WNT7A/B*, and which was also identified as a candidate FOXB2 interactor (29, 41). In contrast, FOXI-exclusive interactors included several transcription cofactors such as BCORL1 and ARID3A (Fig. 6*C*), which may conceivably mediate its specific effects.

Taken together, our data suggest extensive regulation of Wnt pathway–associated genes by FOX transcription factors. We therefore investigated whether evidence for Wnt pathway regulation could be found in public gene expression datasets. Across 12 different studies in which the expression of a single FOX gene in human cells had been altered by gain- or loss-of-function, we did not see a consistent pattern in the regulation of individual Wnt pathway–related genes (Fig. S9*A*). Accordingly, gene set enrichment analyses against a curated list of TCF/LEF target genes (42) only supported Wnt pathway regulation in a few of the datasets (Fig. S9*B*). We previously reported that regulation of Wnt/β-catenin signaling is just one of many functions of FOXQ1 (20); similarly, Gene Ontology analyses of the aforementioned datasets showed that the included FOXs primarily control the expression of non-Wnt pathway–related genes (Supplemental Dataset 1). We conclude that the regulation of Wnt signaling by FOX transcription factors is likely highly context-dependent and that it occurs in parallel with other transcriptional changes within the cell.

## Discussion

Numerous reports have implicated FOX transcription factors in the regulation of Wnt/β-catenin signaling (15), but mainly due to inconsistent methodology, it is difficult to draw overarching conclusions from these earlier studies. Using complementary gain-of-function assays of all human FOXs, we now show that most FOX proteins affect β-catenin/TCF transcriptional activity, TCF/LEF target gene expression, and the regulation of secreted Wnt pathway regulators, including FOX family members that have not been investigated in this context. Moreover, we show that multiple FOX proteins associate with similar transcription cofactor complexes as Tcf7l1 and may thus act as β-catenin–independent regulators of TCF/LEF target genes. Finally, we use these observations to characterize FOXD and FOXI transcription factors as Wnt pathway regulators.

In recent years, there has been a renewed interest in investigating the transcriptional regulation of Wnt/β-catenin pathway target genes, many of which have important functions in development and pathobiology (31). Although it is thought that most of these target genes are controlled primarily by TCF/LEF, other transcription factors such as TBX, CDX, and SOX family members may contribute to the regulation of specific targets (4, 5, 6). FOX transcription factors are similarly known to control Wnt pathway activity in various contexts, and it has been shown that several prototypical TCF/LEF targets including *AXIN2* are differentially regulated by FOXs independently of β-catenin/TCF (20, 21, 29). Our findings expand on these observations and highlight new candidate Wnt pathway regulators within the FOX family across multiple types of cells. For example, we show that FOXO6 affects β-catenin/TCF reporter activity and target gene expression to a similar extent as other FOXO proteins. Although this finding is not entirely surprising considering that FOXO3/4 in particular are well-established Wnt pathway inhibitors (13, 16, 37), the role of FOXO6 remained to be formally established. FOXO6 has distinct subcellular shuttling dynamics and a more restricted expression pattern than other FOXOs (8, 43) and contributes to the pathogenesis of several types of tumors, including breast and colorectal cancer (44, 45). It thus appears worthwhile to explore if FOXO6 inhibits Wnt signaling in human cancers.

We additionally find that class I FOXs are candidate inhibitors of Wnt/β-catenin signaling. It was recently reported that conditional deletion of *Foxi3* increased β-catenin/TCF reporter activity in developing mouse teeth (34). Tooth formation requires tightly regulated Wnt pathway activity (46), suggesting that our identification of FOXIs as Wnt signaling inhibitors is likely physiologically relevant. The mechanism by which FOXIs inhibit Wnt signaling requires further investigation. It has been reported that FOXI1 decreases gastric cancer cell proliferation through the posttranscriptional destabilization of *WNT3A* mRNA (47). However, we find that at least in 293T cells, FOXI1 does not decrease *WNT3A* transcript levels and that it inhibits Wnt signaling in the absence of WNT coreceptors. Our results indicate that FOXIs may instead control β-catenin protein stability, as has been shown for FOXF2 (48).

The identification of FOXDs as candidate Wnt pathway activators is consistent with the recent observation that silencing of FOXD1 lowered β-catenin levels in prostate cancer cells and concomitantly decreased cell proliferation and invasion (33). FOXD1-3 belong to a group of FOX proteins that strongly induce WNT gene expression, and we observed that inhibition of WNT7/RECK signaling was sufficient to block the effect of FOXD1 in TOPflash reporter assays. We have previously shown that FOXB2 controls Wnt pathway activity in prostate cancer cells *via* WNT7/RECK (29), suggesting that these and potentially other FOX family members are functionally redundant in the context of Wnt/β-catenin signaling.

We find that induction of various FOX proteins causes a substantial transcriptional rewiring of the Wnt/β-catenin pathway, which involves the differential regulation of secreted Wnt pathway activators and inhibitors as well as TCF/LEF target genes. While FOX transcription factors can regulate Wnt pathway target genes independently of β-catenin and TCF/LEF (20, 21, 29), it is likely that the parallel induction of Wnt pathway agonists and antagonists triggers feedback loops that reinforce the transcriptional outcome of FOX-dependent Wnt signaling. Additionally, induction of secreted Wnt pathway regulators by FOX transcription factors may allow for the paracrine control of Wnt signaling activity. For example, it has been shown that *FOXL1*-expressing telocytes are an essential source of WNT proteins and R-spondins for intestinal epithelial stem cells (49). Our data suggest that FOXL1 may directly participate in the regulation of Wnt pathway agonists and thereby contribute to normal intestinal homeostasis.

The results of this study are largely based on overexpression, which produces superphysiological abundance and activity levels of FOX transcription factors. In contrast, FOX protein activity *in vivo* is controlled by restricted expression and posttranslational modifications that affect their DNA-binding specificity and the recruitment of critical transcription cofactors (7, 14). Accordingly, it is well established that FOX family members act as regulators or terminal effectors in other conserved cell signaling pathways such as insulin/PKB, hedgehog/GLI, MAP kinase, and TGF-β/SMAD signaling (14), which are known to intersect with the Wnt/β-catenin signaling pathway (50, 51). It is thus likely that FOX transcription factors function as signaling hubs that connect different cell communication cascades through the transcriptional regulation of pathway effectors, which may be particularly relevant for tissue morphogenesis during development.

It is known that the function of FOX proteins can differ considerably between different tissues and cell types. For example, it was recently shown that FOXF2 functions as a Wnt pathway inhibitor in basal-like breast cancer cells but that it activates Wnt signaling in luminal breast cancer (52). The authors attributed these effects to the recruitment of specific transcription cofactors that differentially regulate the expression of WNT genes and receptors. Similarly, FOXQ1 has opposing functions in carcinoma and melanoma cells, which may be mediated by the cell type–dependent recruitment of β-catenin (53). Moreover, it is likely that many FOX family members have more than one mode of action in the Wnt pathway as has been shown for, *e.g.*, FOXM1 (54), FOXQ1 (20), and also FOXF2 (48, 52, 55). Finally, it is known that some FOXs additionally contribute to β-catenin–independent Wnt signaling pathways such as Wnt/planar cell polarity and Wnt/calcium signaling (15), which we did not assess in this study. Thus, the relevance of our findings for different biological contexts necessitates thorough further investigation.

In conclusion, we provide a comprehensive overview of the function of FOX transcription factors in the Wnt/β-catenin signaling pathway. Similar to other transcription factor families, FOX proteins may act as context and gene-specific rheostats of Wnt pathway activity and thereby fine-tune the transcriptional outcome of Wnt signaling. Considering the relevance of both Wnt signaling and FOX transcription factors for human pathobiology, it is likely that these findings are important for cancer biology in particular.

## Experimental procedures

### Cell lines

Authenticated 293T, HCT116, PC3, L, and L/Wnt3a cells were obtained from the German Collection of Microorganisms and Cell Cultures (DSMZ) and the American Type Culture Collection (ATCC). 293T ΔCTNNB1, Penta-KO, and ΔLRP5/6 cells have been described elsewhere (20, 21). MCF-7, HeLa, and SAOS2 cells were kindly provided by Francisca Lottersberger and Per Magnusson (Linköping University), respectively. StemProTM Human Adipose–Derived Stem Cells (mesenchymal stem cell) were purchased from Thermo Fisher Scientific and were cultured in MesenPRO RS medium. Cells were cultured in 1:1 Ham’s F12/RPMI 1640 (PC-3) or Dulbecco’s modified Eagle’s medium (all other cells) with 10% fetal bovine serum, 2 mM glutamine, and 1% penicillin and streptomycin at 37 °C/5% CO_2_. All experiments were performed using low passage cells from mycoplasma-free frozen stocks, as confirmed by analytical qPCR (Eurofins Genomics). Wnt3a and control conditioned media were collected from stably transfected or parental L cells, following the supplier’s guidelines. R-spondin 3 conditioned media were generated by transient transfection of Rspo3ΔC (56) into 293T cells.

### Plasmid construction and transfection

The construction and validation of the FOX transcription factor library used in this study has been described previously (19). The Flag-LEF1 plasmid and WNT7B promoter reporter were described in Moparthi *et al.* (29). For the WNT1 reporter, the WNT7B promoter was replaced by a 1.5 kb fragment of the *WNT1* promoter region. TurboID constructs were prepared by cloning of gene of interest into the N-terminally located TurboID pCS2 flag. All constructs were confirmed by partial sequencing (Eurofins Genomics). Cell transfection was performed using Lipofectamine 2000, Lipofectamine 3000, or Lipofectamine stem transfection reagent (Thermo Fisher Scientific), or jetOPTIMUS (Polyplus Transfection), according to the supplier’s recommendations. Other plasmids used in this study include the following: M50 Super 8x TOPflash and M51 Super 8x FOPflash (18) (Addgene plasmids 12456/12457, deposited by Randall Moon, University of Washington, School of Medicine); Renilla luciferase control plasmid (Addgene plasmid 12179, deposited by David Bartel, Whitehead Institute for Biomedical Research); Flag-LRP6 ΔE1-4 (57) (a gift from Christof Niehrs, Institute of Molecular Biology); and mCherry-Beta-Catenin-20 (Addgene plasmid 55001, deposited by Michael Davidson, Florida State University). In some experiments, cells were additionally treated with GSK3 inhibitor CHIR90221 (Sigma Aldrich), porcupine inhibitor LGK974 (Cayman Chemicals), or proteasomal inhibitor bortezomib (a gift from Padraig Dárcy, Linköping University). siRNAs against RECK (#1) and WNT7B (#3) were purchased from Integrated DNA Technologies (IDT) and validated previously (29). Scrambled siRNA control was from Thermo Fisher Scientific.

### Reporter assays

For reporter assays, cells were seeded on a 96-well plate and transfected with 50 ng firefly reporter, 5 ng renilla control, and 10 ng of the plasmid of interest in each well. Where indicated, 6 h after transfection, cells were treated with control, Wnt3a (standard dilution 1:5) or R-spondin 3 conditioned media (standard dilution 1:1000), or a combination of both (indicated as W/R). The dual luciferase assay was conducted as described previously (58) with few changes. Briefly, after overnight incubation, cells were lysed in passive lysis buffer (25 mM Tris, 2 mM DTT, 2 mM EDTA, 10% (v/v) glycerol, 1% (v/v) Triton X-100, (pH 7.8)) and agitated for 10 min. Lysates were transferred to a flat bottomed 96-well luminescence assay plate. Firefly luciferase buffer (200 μM D-luciferin in 200 mM Tris–HCl, 15 mM MgSO4, 100 μM EDTA, 1 mM ATP, 25 mM DTT, pH 8.0) was added to each well and the plate was incubated for 2 min at room temperature. Luciferase activity was measured using Spark10 (Tecan) or a SpectraMax iD3 Multi-Mode Microplate Reader (Molecular Devices). Next, Renilla luciferase buffer (4 μM coelenterazine-h in 500 mM NaCl, 500 mM Na2SO4, 10 mM NaOAc, 15 mM EDTA, 25 mM sodium pyrophosphate, 50 μM phenyl-benzothiazole, pH 5.0) was added to the plate and luminescence was measured immediately. Data were normalized to the Renilla control values, performed in triplicate.

### TaqMan qPCR array

qPCR array experiments were performed using custom 384-well TaqMan Gene Expression Array Cards (Thermo Fisher Scientific) containing inventoried probes for 48 gene targets, including three controls (18s rRNA, GAPDH, HPRT1). Data were normalized to the geometric mean of the controls. Experiments were done in 293T cells pretreated with 5 ng/ml recombinant human R-spondin 3 (R&D Systems). Experiments were performed with three biological replicates and two technical replicates. For control of R-spondin effects, we included two untreated, empty vector transfected samples. Data were acquired on a QuantStudio 7 Flex Real-Time PCR system (Thermo Fisher Scientific). All gene expression data averaged from the technical replicates, excluding the untreated controls, can be found in the supplemental materials.

### Quantitative real-time PCR

qPCR was performed using standard protocols. Briefly, RNA was extracted using a Qiagen RNeasy mini kit and reverse transcribed using a high-capacity cDNA reverse transcription kit (Thermo Fisher Scientific). Complementary DNA was amplified using validated custom primers, with SYBR green dye. Data were acquired on Bio-Rad CFX96 touch thermocycler and normalized to HPRT1 control.

### Immunoblotting and immunoprecipitation

Cells were harvested in PBS and lysed in lysis buffer (1% NP-40 in PBS with 1× protease inhibitor cocktail). Lysates were boiled in Laemmli sample buffer with 50 mM DTT, separated on 10% polyacrylamide gels (Bio-Rad), transferred onto nitrocellulose membranes, and incubated in blocking buffer (LI-COR). Primary antibodies were detected using near-IR fluorophore-labeled secondary antibodies (LI-COR). Blots were scanned on a LI-COR CLx imager. Antibodies used in this study are as follows: mouse anti-Flag M2 (F3165) and anti-Flag affinity gel (A2220) from Sigma Aldrich; rabbit anti-HSP70 (AF1663) from R&D Systems; rabbit anti-non phospho (Active) β-catenin (8814) from Cell Signaling Technology; mouse anti-FOXI (TA800145) from Thermo Fisher Scientific.

### TurboID sample preparation

The labeling and sample preparation of TurboID experiments was performed as described previously (20, 24). Briefly, N-terminal TurboID-FOX proteins and TurboID plasmids were transiently transfected into 293T cells using jetOPTIMUS (Polyplus Transfection). After 21 h of transfection, cells were treated with 50 μM biotin and incubated for 3 h at 37 °C, 5% CO_2_. Cells were surface washed with ice-cold PBS for three times to remove excess biotin and then harvested centrifuging at 1500 rpm for 15 min. Cells were washed thrice with ice-cold PBS buffer by centrifugation to remove any remaining biotin. Cells were lysed in RIPA buffer containing 1x protease inhibitor cocktail for 15 min on ice. Prewashed streptavidin beads (GE Healthcare) were added to the cell lysate and incubated overnight at 4 °C with end-over-end rotation. The beads were washed once with 1 ml of RIPA buffer, once with 1 ml of 1 M KCl, once with 1 ml of 0.1 M Na_2_CO_3_, once with 1 ml of 2 M urea in 10 mM Tris–HCl (pH 8.0), and twice with 1 ml RIPA lysis buffer. The beads then transferred to new Eppendorf tube and washed twice with 50 mM Tris–HCl buffer (pH 7.5) and 2 M urea/50 mM Tris (pH 7.5) buffer. Beads were incubated with 0.4 μg of trypsin (Thermo Fisher) in 2 M urea/50 mM Tris containing 1 mM DTT for 1 h at 25 °C with end-over-end rotation. After incubation, the supernatant was collected and the beads were washed twice with 60 μl of 2 M urea/50 mM Tris buffer (pH 7.5) and the washes were combined with the collected supernatant. The supernatant was reduced with 4 mM DTT for 30 min at 25 °C with end-over-end rotation. The samples were alkylated with 10 mM iodoacetamide for 45 min in the dark at 25 °C with end-over-end rotation. For the complete digestion of the sample, an additional 0.5 μg of trypsin was added and incubated at 25 °C overnight with end-over-end rotation. After overnight digestion, the samples were desalted with C18 Thermo Fisher Scientific pipette tips and then dried with vacuum centrifuge.

### Mass spectrometry data acquisition and analysis

TurboID samples were analyzed by mass spectrometry, using an Easy nano LC 1200 system interfaced with a nano Easy-Spray ion source (Thermo Fisher Scientific) connected Q-Exactive HF Hybrid Quadrupole-Orbitrap Mass Spectrometer (Thermo Fisher Scientific). The peptides were loaded on a precolumn (Acclaim PepMap 100, 75 μm × 2 cm, Thermo Fisher Scientific) and the chromatographic separation was performed using an EASY-Spray C18 reversed-phase nano LC column (PepMap RSLC C18, 2 μm, 100A 75 μm × 25 cm, Thermo Fisher Scientific). The nano LC was operating at 300 nl/min flow rate with a gradient (6–40% in 95 min and 5 min hold at 100%) solvent B (0.1% (v/v) formic acid in 100% acetonitrile) in solvent A (0.1% (v/v) formic acid in water) for 100 min. Separated peptides were electrosprayed and analyzed using a Q-Exactive HF mass spectrometer (Thermo Fisher Scientific), operated in positive polarity in a data-dependent mode. Full scans were performed at 120,000 resolutions at a range of 380 to 1400 m/z. The top 15 most intense multiple charged ions were isolated (1.2 m/z isolation window) and fragmented at a resolution of 30,000 with a dynamic exclusion of 30.0 s. Raw data were processed by Proteome Discover 2.0 (Thermo Fisher Scientific) searching against the *Homo sapiens* UniProt database (release 2019-12-16) with Sequest HT search engine. The search parameters were as follows: taxonomy: *H. sapiens*; enzymes; trypsin with two missed cleavages, no variable modifications; fixed modification: Carbamidomethyl; Peptide Mass Tolerance, 10 ppm; MS/MS Fragment Tolerance, 0.02 Da. Quantification of the analyzed data were performed with Scaffold 5.1.0 (Proteome Software Inc, https://www.proteomesoftware.com/products/scaffold-5) using total spectral count. Protein and peptide identifications were accepted if they could be established at greater than 95 and 90% probability, respectively and if protein identification contained at least two identified peptides.

### Colony formation assays

HCT116 were seeded on 6-well plates at a density of 1000 cells after 24 h of transfection. Cells were grown for 14 days. At the end of the experiment, cells were washed with PBS and stained with 2% methylene blue in 50% methanol. Stained plates were photographed and colonies were counted manually. Colonies with less than 50 cells were excluded.

### *In silico* transcription factor–binding analysis

For *in silico* prediction of transcription factor binding to Wnt pathway–related gene promoters, we retrieved positional weight matrices for 27 human FOX family proteins and all four human TCF/LEF proteins from the JASPAR 2022 database (59) (Table S2). For single transcription factor–binding prediction, we obtained the human promoter sequences (range: −499 to +100 from the transcription start site) for all Wnt pathway genes included in the qPCR array, as well as all Frizzled family receptors, LRP5/6, and Wnt pathway inhibitors NOTUM, SOST, and TIKI/TRABD2A from the Eukaryotic Promoter Database (60) or Ensembl (61). Sequences were aligned using the searchSeq function in R package TFBSTools v1.34.0 (62), with a minimum score of 90%. Empirical *p*-values were determined using the *p*-values function implemented in TFBSTools with “sampling” option. We additionally analyzed potential FOX/TCF/LEF co-occupancy of TCF/LEF target gene promoters. For this, we retrieved the extended promoter sequence (range: −999 to +100 from the transcription start site) for the target genes included in our qPCR array, as well as their mouse orthologs, from the Eukaryotic Promoter Database. Co-occupancy was predicted using MCAST v5.5.1 (63) with the following options: *p*-value <0.0005, motif spacing ≤50 bp, E-value <10. Because the number of mouse FOX motifs curated in the JASPAR database is limited, and the forkhead box domain of most FOX proteins is highly conserved across species, we analyzed mouse promoters using the human transcription factor motifs to allow for easier comparison of the results.

### Data quantification and analysis

Most statistical analyses were performed in R v4.2.0 (64) and were based on three or more biological replicates per condition, that is, independent samples receiving the same treatment from one or more individual experiments. Statistical tests are indicated in the figure legends. Hierarchical clustering was done using the k-means and hclust functions implemented in R package ComplexHeatmaps v2.12.1 (65), with a fixed seed and 1000 iterations. Apart from data in Figure 1, optimal cluster numbers were determined using the Silhouette method (66) implemented in R package factoextra v1.0.7 (https://rpkgs.datanovia.com/factoextra/index.html). Proteomics data were processed using SAINTexpress, CRAPome, and ProHits-*viz.* (67, 68, 69), as described previously (20), including data from Moreira *et al.* (30) following mouse-to-human gene name conversion. Normalized transcript counts for the following public bulk RNA-seq datasets were obtained through the GREIN repository (70): GSE108500, GSE126564, GSE142221, GSE151059, GSE160001, GSE169334, GSE174462, GSE182515, GSE64513, GSE81084, GSE86956. Gene Ontology and enrichment analyses of proteomics data were performed using R package clusterProfiler v4.4.4 (71). Gene set enrichment analyses against a curated list of TCF/LEF target genes (42) were performed using the GSEA function implemented in clusterProfiler. CORUM protein complexes v4.1 have been described in Tsitsiridis *et al.* (28). Proteomics data in Fig. S5*A* are from Li *et al.* (26). Only hits with a spectral count >1 were included in the analysis. Bar graphs depict group means with SD and individual data points.

## Data availability

TOPflash and Taqman qPCR array data can be found in the supplemental materials. TurboID proteomics data have been deposited to the ProteomeXchange Consortium *via* the PRIDE (72) partner repository with the dataset identifier PXD038811 and 10.6019/PXD038811. All other raw data generated in this study, as well as R scripts to reproduce the results presented here, will be provided upon request.

## Supporting information

This article contains supporting information (20, 26, 28, 29, 30).

## Conflict of interest

The authors declare that they have no conflicts of interest with the contents of this article.
