# Supplementary material for: FOX transcription factors are common regulators of Wnt/β-catenin–dependent gene transcription
Source: J Biol Chem. 2023 Apr 1;299(5):104667. doi: 10.1016/j.jbc.2023.104667 (PMC10193241; doi:10.1016/j.jbc.2023.104667)
Supplement: Supporting Figures S1–S9 and Tables S1 and S2 [file mmc1.docx]

**Title: FOX transcription factors are common regulators of Wnt/β-catenin-dependent gene transcription**

**Authors**: Lavanya Moparthi, Stefan Koch

This document contains the following supporting information:

Supplemental figure 1: TOPflash assay controls.

Supplemental figure 2: qPCR array controls.

Supplemental figure 3: *In silico* transcription factor binding prediction.

Supplemental figure 4: FOX proteins regulate secreted Wnt pathway inhibitors.

Supplemental figure 5: FOX proteins and Tcf7l1 share interacting protein complexes.

Supplemental figure 6: FOXDs and FOXIs are Wnt pathway regulators.

Supplemental figure 7: FOXIs regulate β-catenin stability.

Supplemental figure 8: FOXD1 and FOXI1 have distinct interactors.

Supplemental figure 9: Analysis of public datasets.

Supplemental table 1: Overview of studies linking FOX transcription factors to Wnt signaling, with the methods used to support this conclusion.

Supplemental table 2: List of positional weight matrix (PWM) identifiers used for *in silico* analyses.

**
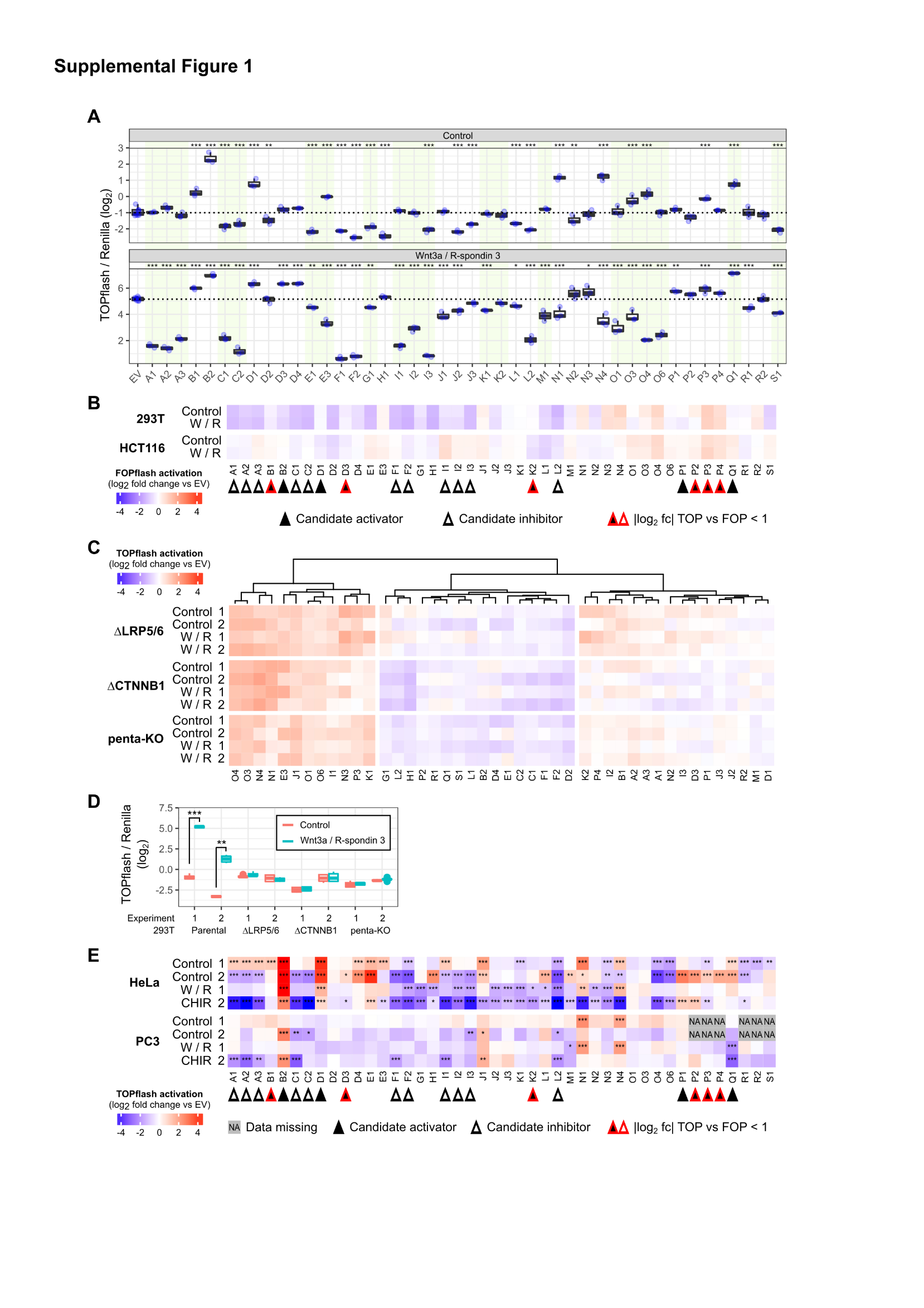
**

**Supplemental figure 1. TOPflash assay controls.** (**A**) Representative TOPflash results with individual data points, corresponding to 293T experiment 1 in Fig. 1B. Data are displayed as the Firefly to Renilla luciferase activity without additional normalization to empty vector (EV) control. Statistical significance was determined by Dunnett’s post-hoc test following one-way ANOVA. (**B**) Activity assay using the control reporter plasmid FOPflash, which contains scrambled TCF binding sites. Data were normalized to EV control. Where indicated, cells were treated with Wnt3a / R-spondin 3 (W/R) conditioned media. Candidate activators and inhibitors were defined in Fig. 1D. For candidates marked with a red outline, we observed a smaller than 2-fold difference in TOPflash versus FOPflash regulation. (**C**) TOPflash assay in 293T lacking the Wnt co-receptors LRP5/6 (ΔLRP5/6), β-catenin (ΔCTNNB1), or β-catenin and all TCF/LEF proteins (penta-KO). (**D**) TOPflash results from all EV-transfected cells used in the previous assays, following Wnt3a / R-spondin 3 treatment. (**E**) TOPflash assay in HeLa (cervical cancer) and PC3 (prostate cancer) cells. Where indicated, cells were treated with Wnt3a / R-spondin 3 (W/R) conditioned media or 5 µM GSK3 inhibitor CHIR99021. Statistical significance in panels A and E was determined by Dunnett’s post-hoc test following one-way ANOVA. Data in D were analyzed using an unpaired Welch’s t-test with Bonferroni-Hochberg correction for multiple testing. *** P<0.001, ** P<0.01, * P<0.05.


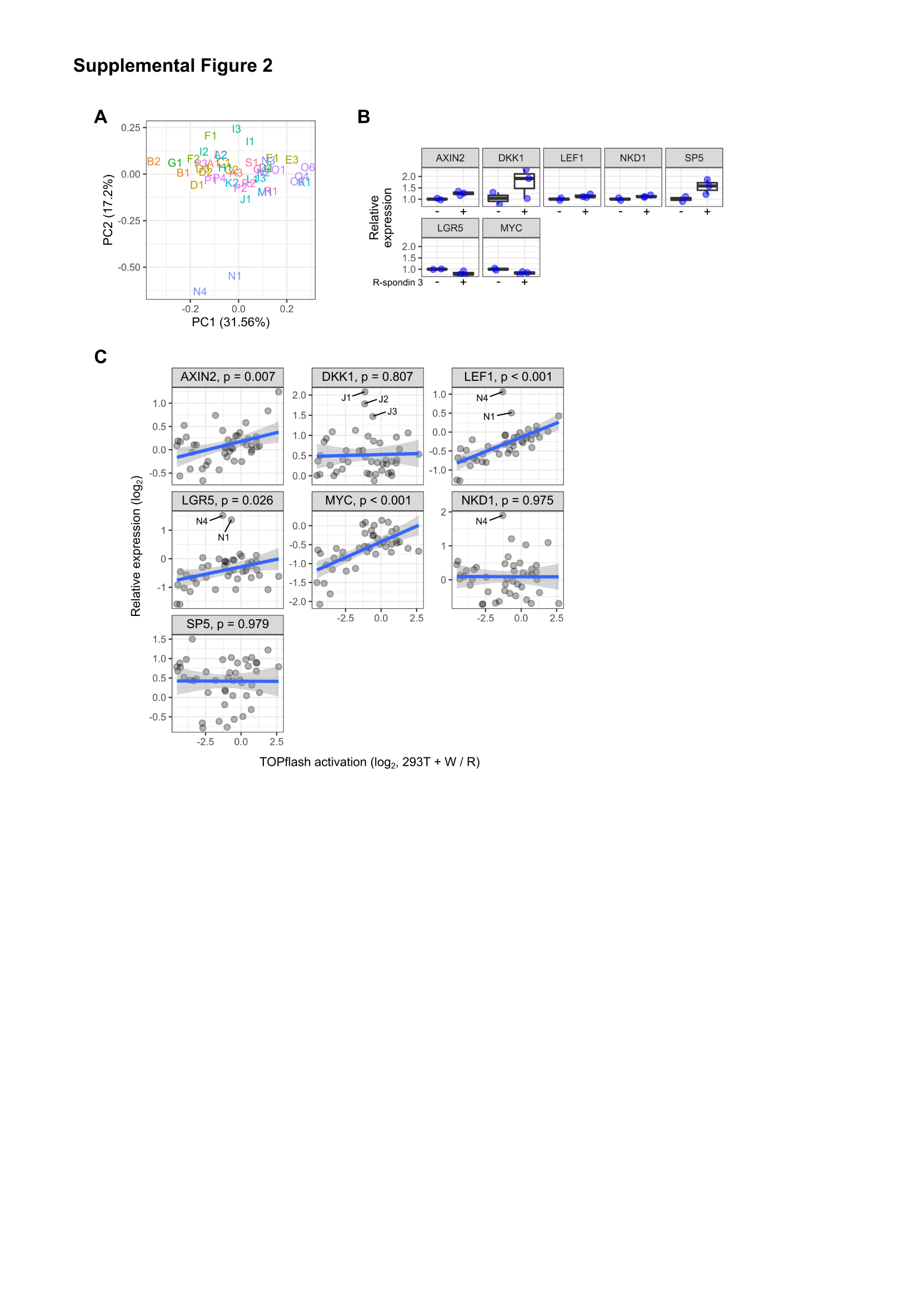


**Supplemental figure 2: qPCR array controls.** (**A**) Principal component plot based on expression changes of all genes included in the qPCR array, following normalization to housekeeping controls. (**B**) Expression changes of Wnt target genes included in the array following treatment with 5 ng/ml recombinant human R-spondin 3 (n=3) versus untreated control (n=2). All cells were transfected with empty vector. (**C**) Correlation analyses of Wnt target gene expression changes against averaged results from TOPflash assays in Fig. 1B. Notable outliers are highlighted. P-values are based on Spearman’s rank correlation.

**
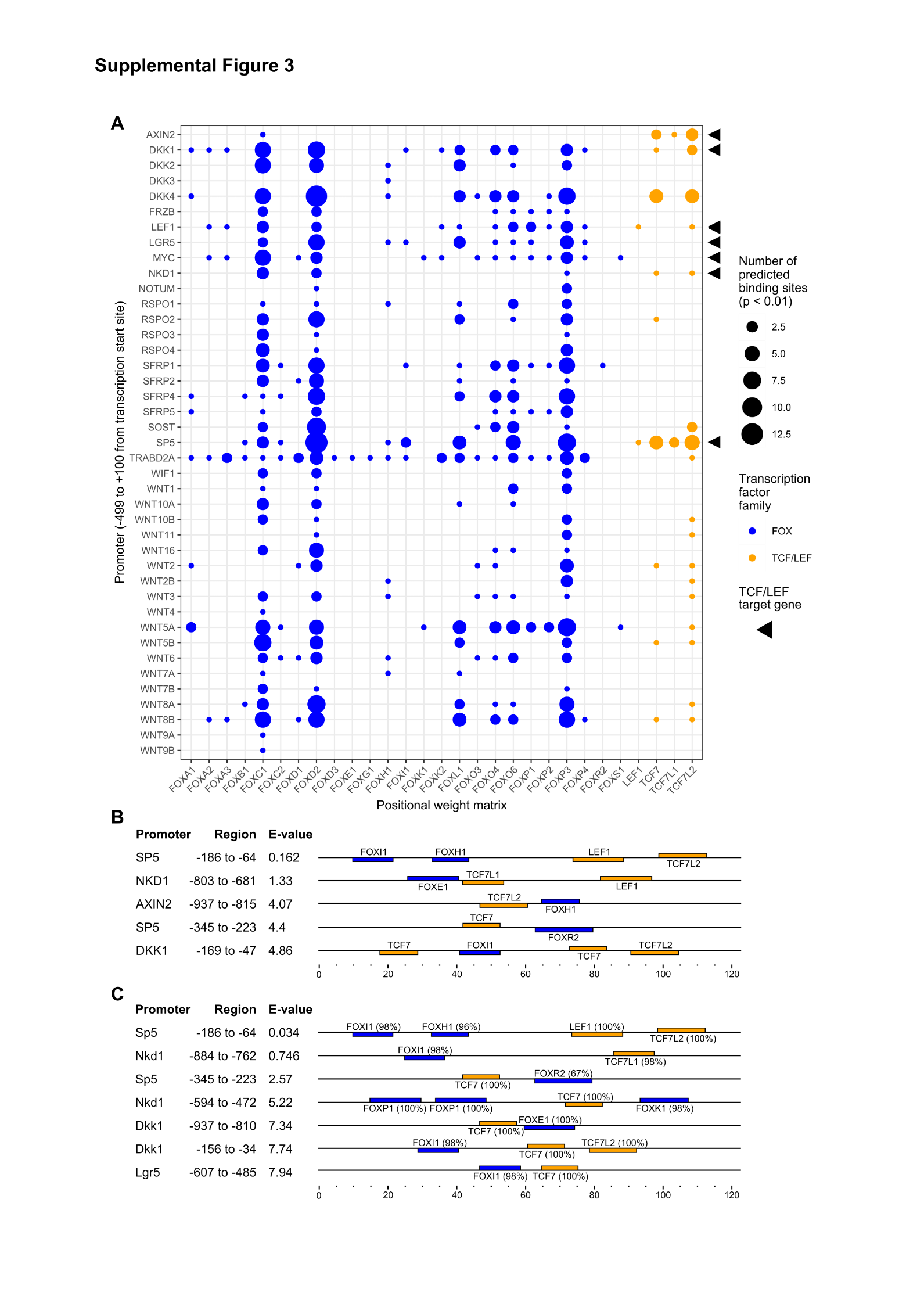
**

**Supplemental figure 3: *In silico* transcription factor binding prediction.** (**A**) Predicted number of binding sites of the indicated human FOX and TCF/LEF proteins in Wnt pathway-related human gene promoters. (**B**) Analysis of potential transcription factor co-occupancy on human TCF/LEF target gene promoters. The schematic on the right illustrates the orientation and distance of identified transcription factors. (**C**) Analysis of potential transcription factor co-occupancy on mouse TCF/LEF target gene promoters. Analyses were performed with human positional weight matrices. Numbers in parentheses indicate the amino acid sequence identity of the human transcription factor DNA-binding domain with its mouse ortholog.


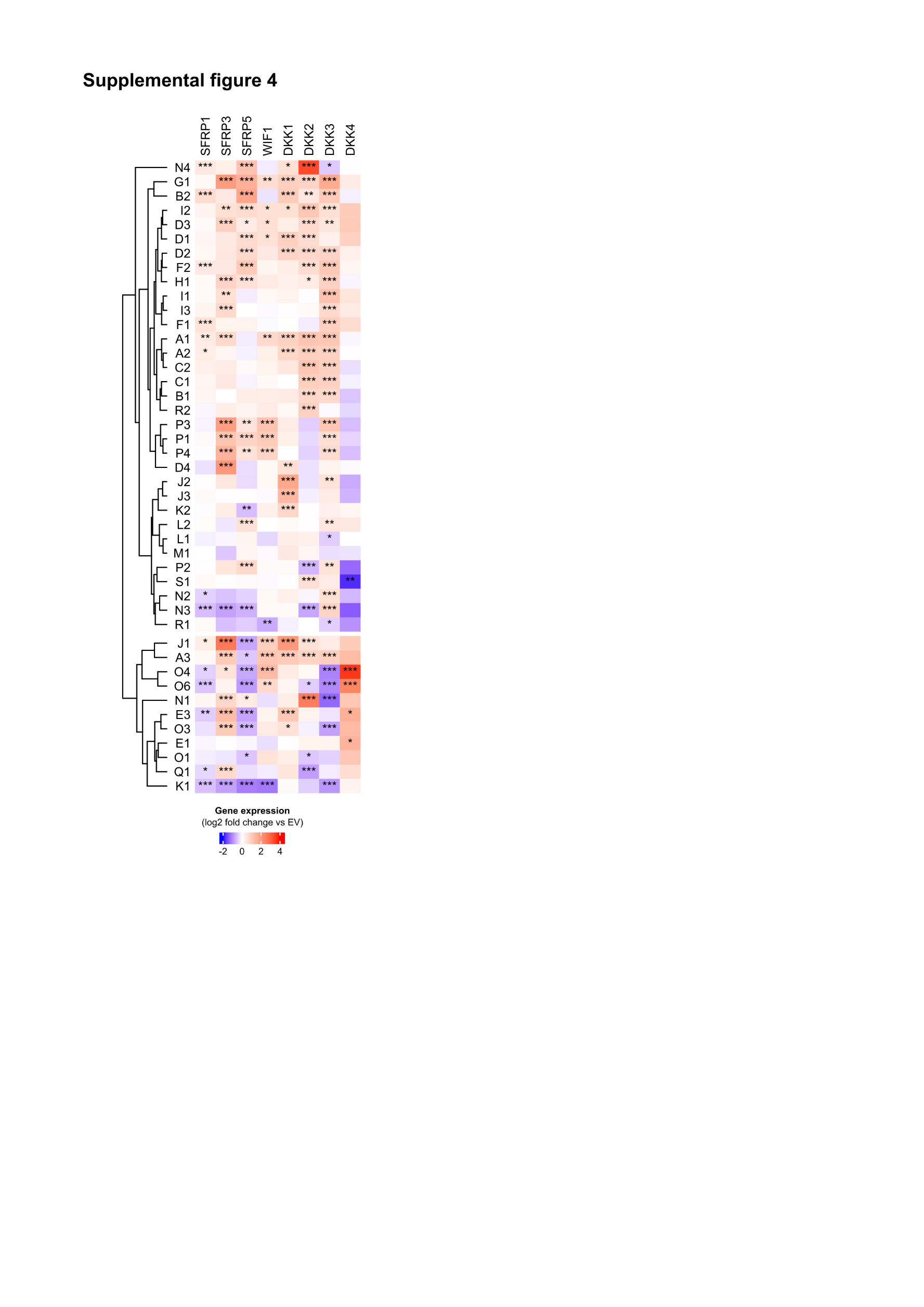


**Supplemental figure 4: FOX proteins regulate secreted Wnt pathway inhibitors.** qPCR array of secreted Wnt pathway inhibitors in 293T treated with 5 ng/ml recombinant human R-spondin 3. Each cell represents the average of three biological replicates, normalized to empty vector (EV) control. Note that SFRP2 was beyond the limit of detection in most samples, and was therefore omitted from this analysis. Data for DKK1 are repeated from Wnt target gene analyses in Fig. 1C. Data were analyzed using Dunnett’s post-hoc test against EV following one-way ANOVA (*** P<0.001, ** P<0.01, * P<0.05).


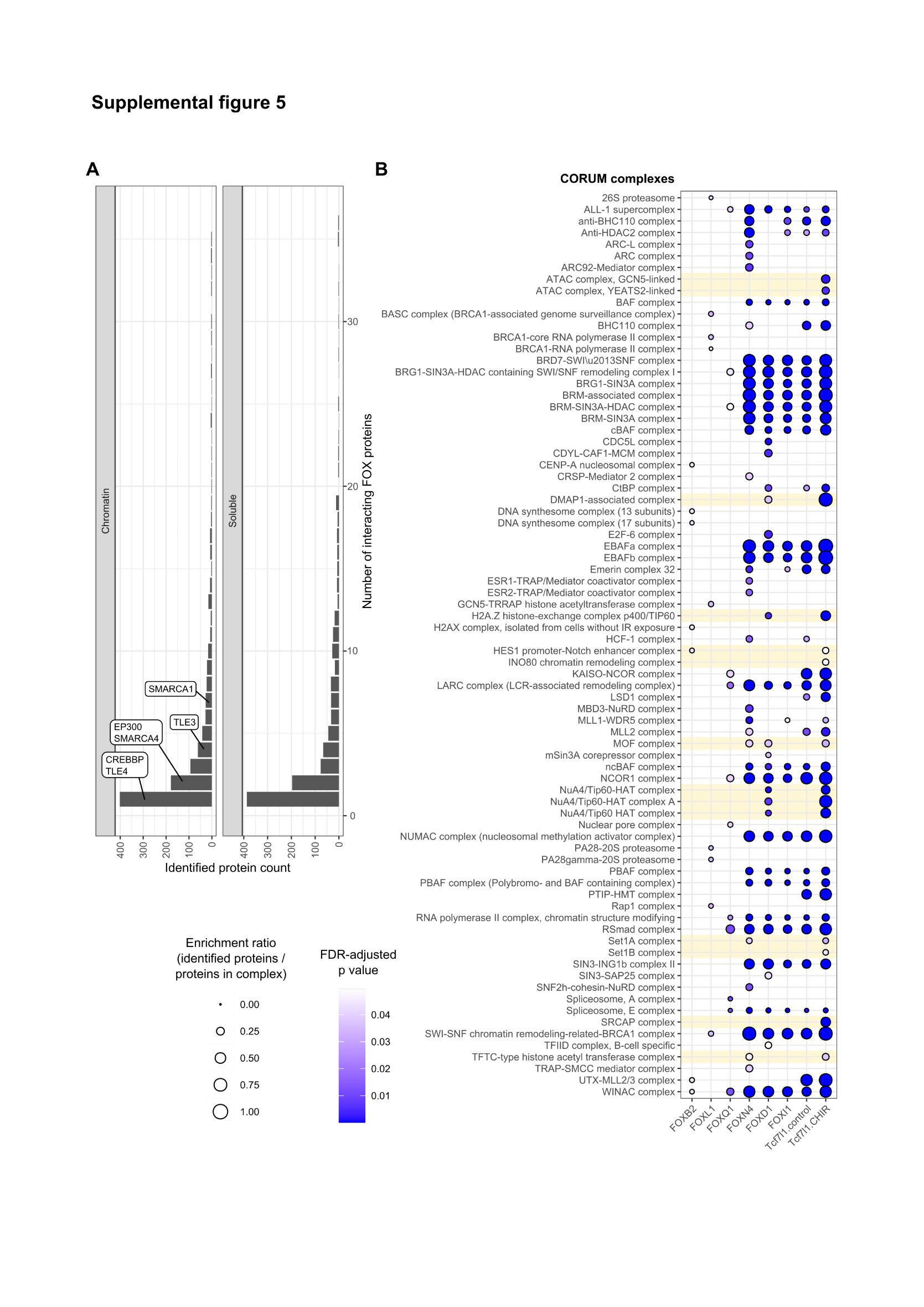


**Supplemental figure 5: FOX proteins and Tcf7l1 share interacting protein complexes.** (**A**) Candidate FOX interactors identified by Li et al.(1) using co-immunoprecipitation / mass spectrometry following expression of 36 FOX family members in 293T. Data are separated into chromatin and soluble sample fractions. Some interactors of interest that were also found in our TurboID data are highlighted. (**B**) Enrichment analysis against all human protein complexes curated in the CORUM database.(2) All complexes that were significantly enriched in at least one FOX sample or Tcf7l1 are shown. Results include proteomics data from earlier BioID-FOXB2, TurboID-FOXQ1, and BioID-Tcf7l1 studies.(3-5) Note that no enriched complexes were identified for FOXG1. Complex names were taken from the CORUM resource as-is, and contain broadly overlapping complexes. Yellow highlighting indicates protein complexes that are associated with Tcf7l1 after CHIR treatment. FDR, false discovery rate.


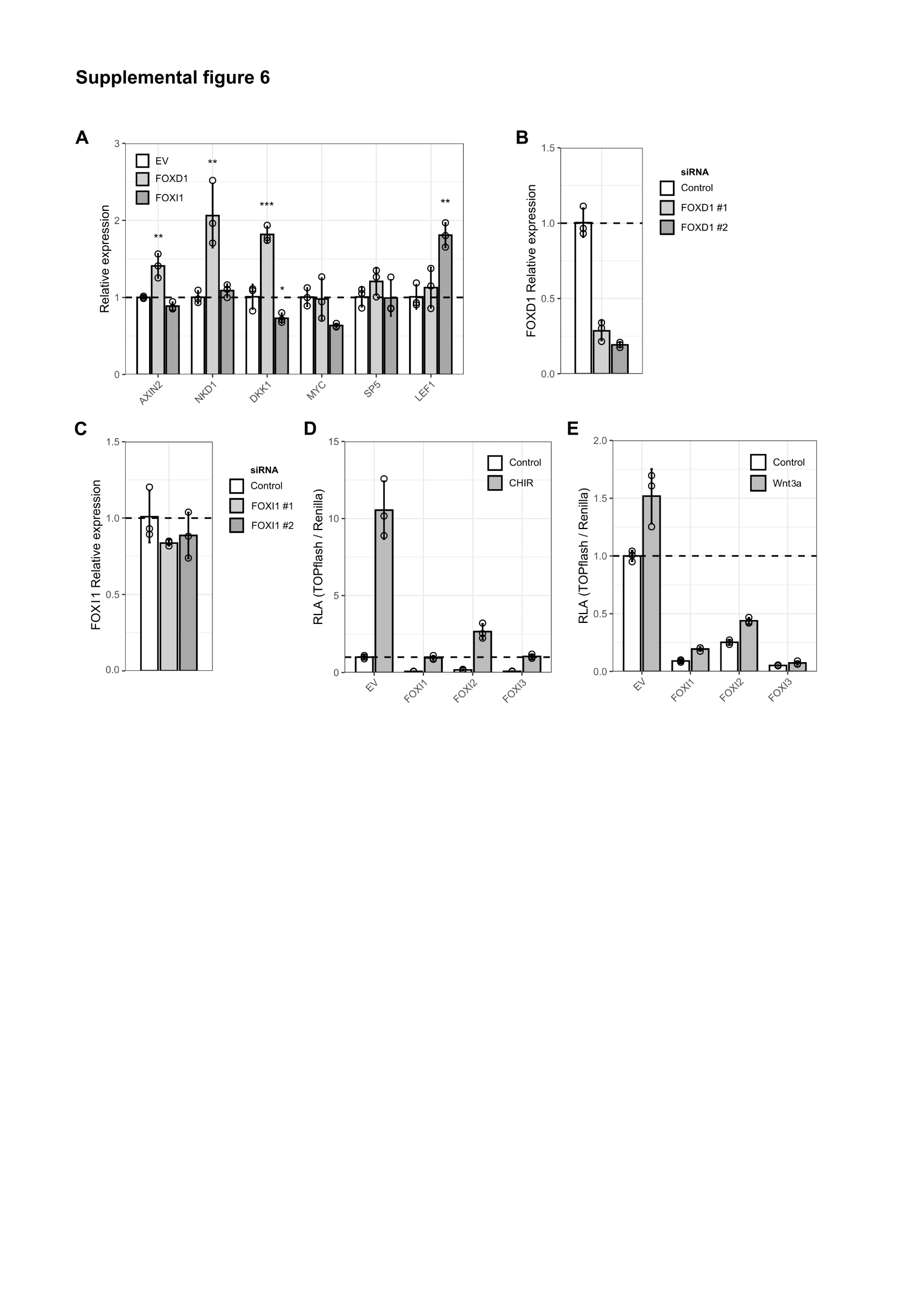


**Supplemental figure 6: FOXDs and FOXIs are Wnt pathway regulators.** (**A**) qPCR analysis of TCF/LEF target genes in HeLa cells following FOXD1 or FOXI1 overexpression. (**B, C**) qPCR analysis of (**B**) *FOXD1* or (**C**) *FOXI1* expression in 293T cells following RNA interference with the indicated siRNAs. (**D**) TOPflash assay in HCT116 following treatment with 5 µM GSK3 inhibitor CHIR99021. (**E**) TOPflash assay in 293T following β-catenin overexpression. Where indicated, cells were treated with Wnt3a conditioned media. Data in panel A were analyzed using Dunnett’s post-hoc test against empty vector (EV) following one-way ANOVA (*** P<0.001, ** P<0.01, * P<0.05). RLA, relative luciferase activity.


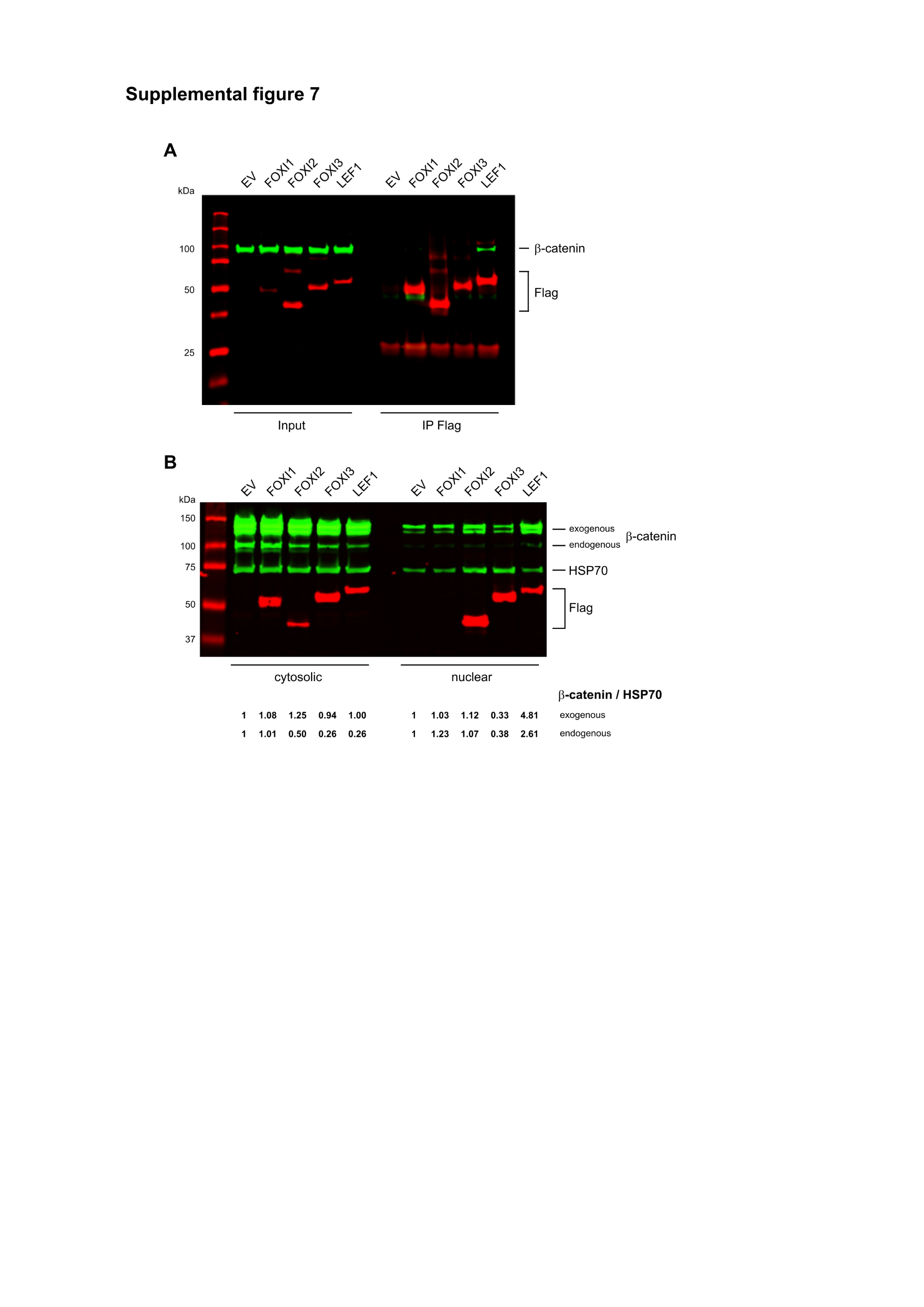


**Supplemental figure 7: FOXIs regulate β-catenin stability.** (**A**) Co-immunoprecipitation assay in HCT116. The indicated proteins were precipitated using anti-Flag agarose. LEF1 was included as a positive control. (B) Immunoblot of β-catenin levels in subcellular fractions of HCT116 cells expressing β-catenin-mCherry. Numbers below the blot indicate the relative β-catenin level normalized to HSP70 housekeeping control and empty vector (EV) levels.


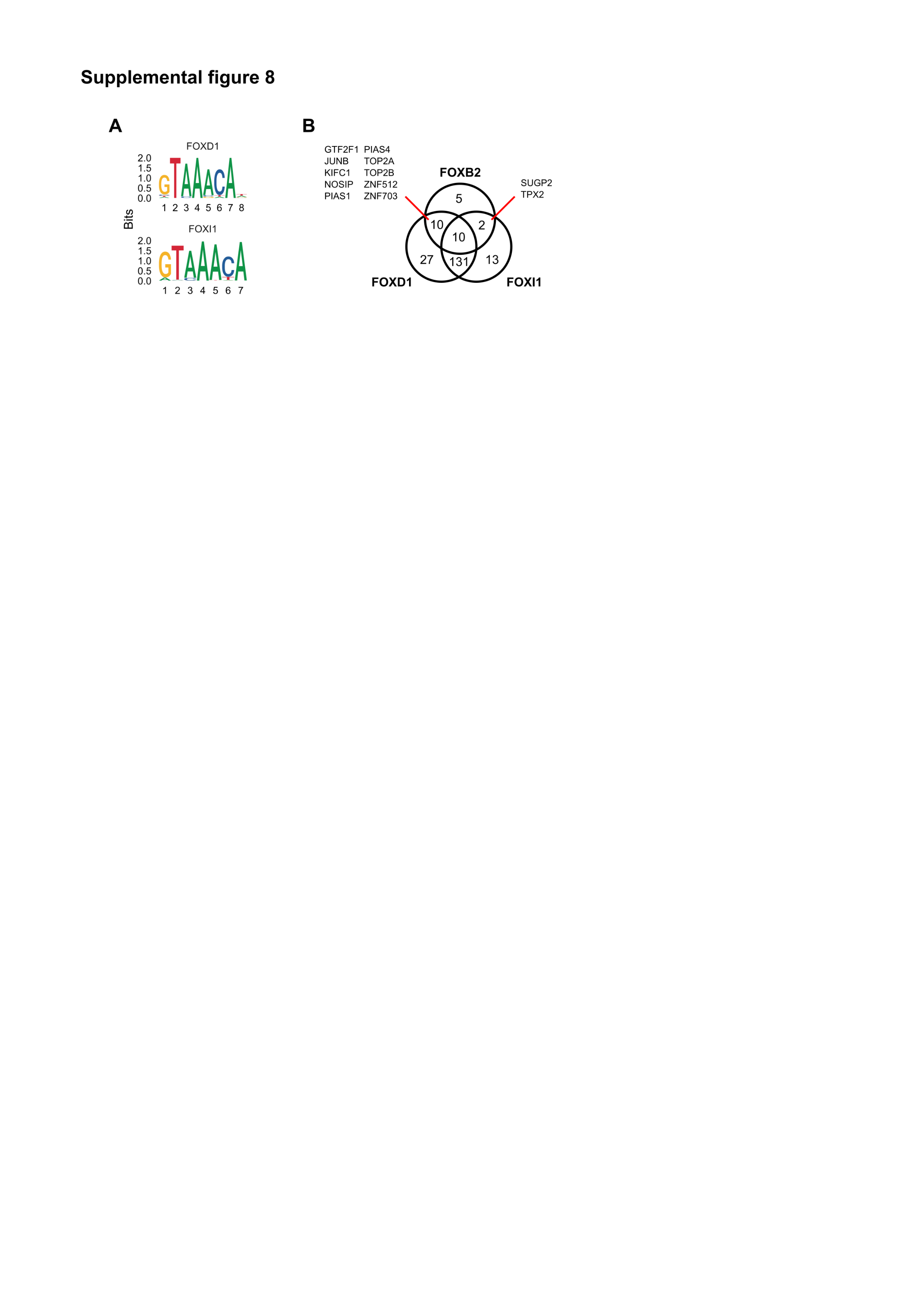


**Supplemental figure 8: FOXD1 and FOXI1 have distinct interactors.** (**A**) Positional weight matrices of FOXD1 and FOXI1. (**B**) Shared interactors between FOXD1, FOXI1, and FOXB2, based on proximity proteomics.


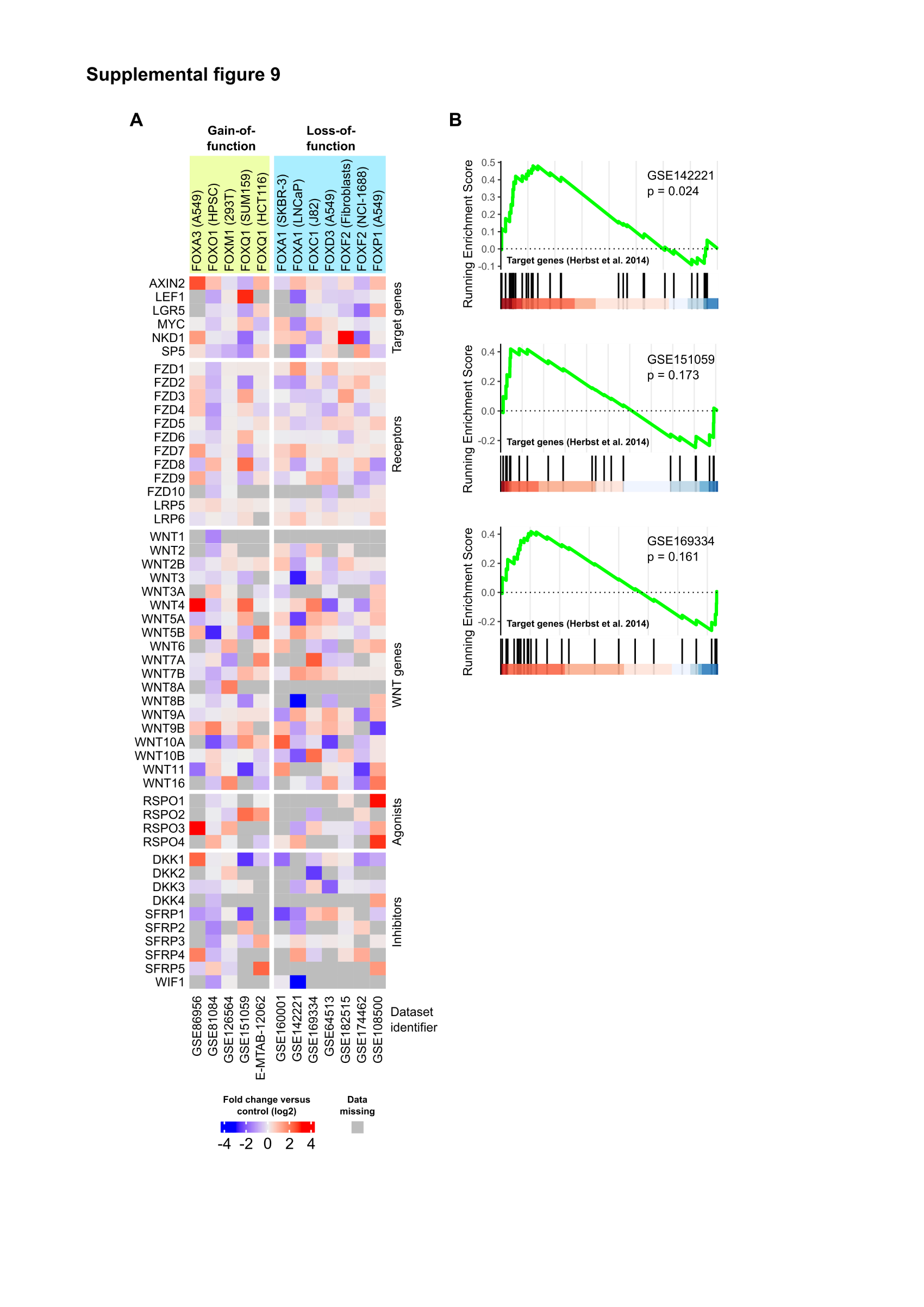


**Supplemental figure 9: Analysis of public datasets.** (**A**) Heatmap of expression changes in Wnt pathway-related genes following FOX gain or loss-of-function in the indicated RNA-seq datasets. Names in parentheses are the cell model used in the study. (B) Gene set enrichment analyses in selected datasets against a set of curated TCF/LEF target genes.

**Supplemental table 1: Overview of studies linking FOX transcription factors to Wnt signaling, with the methods used to support this conclusion.**

| **FOX family member** | **Reference PMID**^a^ | **Wnt pathway activator / inhibitor** | **Evidence for activity in Wnt/β-catenin signaling** | | | | | | | | |
| --- | --- | --- | --- | --- | --- | --- | --- | --- | --- | --- | --- |
|  |  |  | **Wnt reporter assay** | **Target gene expression** | **Target protein levels** | **beta-catenin protein levels** | **beta-catenin nuclear translocation** | **beta-catenin binding** | **TCF/LEF binding** | **Target gene promoter binding** | **Genetic** |
| A1 | 34531670 | Inhibitor |  |  |  | x |  |  |  |  |  |
| A2 |  |  |  |  |  |  |  |  |  |  |  |
| A3 |  |  |  |  |  |  |  |  |  |  |  |
| B1 |  |  |  |  |  |  |  |  |  |  |  |
| B2 | 31611391 | Activator | x | x |  |  |  |  |  |  |  |
| C1 | 33987183 | Activator | x |  |  |  | x | x |  |  |  |
|  | 34427817 | Activator |  |  |  | x | x |  |  |  |  |
|  | 30189871 | Activator |  |  |  | x |  |  |  |  |  |
| C2 | 23645207 | Activator | x |  |  |  |  |  |  |  |  |
| D1 | 35738443 | Activator |  |  | x | x |  |  |  |  |  |
| D2 |  |  |  |  |  |  |  |  |  |  |  |
| D3 |  |  |  |  |  |  |  |  |  |  |  |
| D4 |  |  |  |  |  |  |  |  |  |  |  |
| E1 | 30793770 | Activator |  |  |  | x |  |  |  |  |  |
| E3 |  |  |  |  |  |  |  |  |  |  |  |
| F1 | 16439479 | Inhibitor |  |  |  |  | x |  |  |  | x |
| F2 | 16439479 | Inhibitor |  |  |  |  | x |  |  |  | x |
|  | 23376422 | Inhibitor |  |  | x | x | x |  |  |  | x |
|  | 29374064 | Inhibitor | x | x | x | x | x |  |  |  |  |
|  | 35668101 | Inhibitor | x |  |  |  | x |  |  |  |  |
|  | 35660418 | Both (cell type dependent) | x |  |  |  | x |  |  |  |  |
| G1 | 31771611 | Activator | x |  |  |  | x | x | x | x |  |
|  | 33015737 | Activator |  |  |  | x |  |  |  |  |  |
|  | 19386266 | Inhibitor |  |  |  |  |  |  |  |  | x |
| H1 | 26770350 | Activator |  | x | x | x |  |  |  |  |  |
|  | 34090445 | Activator | x |  | x | x |  |  |  |  |  |
|  | 32650116 | Activator |  | x |  |  |  |  |  |  |  |
| I1 | 28358374 | Inhibitor |  |  | x |  |  |  |  |  |  |
| I2 |  |  |  |  |  |  |  |  |  |  |  |
| I3 |  |  |  |  |  |  |  |  |  |  |  |
| J1 | 28209947 | Activator |  |  | x |  | x |  |  |  |  |
| J2 |  |  |  |  |  |  |  |  |  |  |  |
| J3 |  |  |  |  |  |  |  |  |  |  |  |
| K1 | 25805136 | Activator | x | x |  |  |  |  |  |  |  |
| K2 | 25805136 | Activator | x | x |  |  |  |  |  |  | x |
| L1 | 11555641 | Inhibitor |  |  |  |  | x |  |  |  | x |
| L2 |  |  |  |  |  |  |  |  |  |  |  |
| M1 | 22014570 | Activator | x |  | x |  | x | x |  | x |  |
|  | 26912724 | Activator | x |  | x |  |  | x |  | x |  |
|  | 35695863 | Activator |  | x |  | x |  | x |  |  |  |
|  | 34911926 | Activator |  |  | x |  |  |  |  |  |  |
|  | 35799265 | Activator |  |  | x | x |  |  |  |  |  |
| N1 |  |  |  |  |  |  |  |  |  |  |  |
| N2 |  |  |  |  |  |  |  |  |  |  |  |
| N3 | 28039460 | Inhibitor | x |  | x |  |  | x |  |  |  |
|  | 32619584 | Inhibitor |  |  | x | x |  |  |  |  |  |
| N4 |  |  |  |  |  |  |  |  |  |  |  |
| O1 | 19896444 | Inhibitor | x |  |  |  |  |  |  |  |  |
|  | 26344693 | Inhibitor | x |  |  | x |  |  |  |  |  |
| O3 | 18250171 | Inhibitor | x | x |  |  |  |  |  |  |  |
|  | 19896444 | Inhibitor | x |  |  |  |  |  |  |  |  |
|  | 25578861 | Inhibitor | x | x |  | x |  |  |  |  |  |
|  | 35795985 | Inhibitor |  |  |  | x |  |  |  |  |  |
| O4 | 18250171 | Inhibitor | x | x |  |  |  |  |  |  |  |
|  | 35805009 | Inhibitor |  |  | x | x |  | x |  |  |  |
| O6 |  |  |  |  |  |  |  |  |  |  |  |
| P1 | 25650440 | Activator | x | x |  |  |  | x | x | x | x |
| P2 |  |  |  |  |  |  |  |  |  |  |  |
| P3 | 28716029 | Activator | x | x | x |  |  | x | x |  |  |
| P4 | 34590150 | Activator |  |  | x |  |  |  |  |  |  |
| Q1 | 32943107 | Activator | x |  |  |  | x |  |  |  |  |
| R1 |  |  |  |  |  |  |  |  |  |  |  |
| R2 | 29634928 | Activator |  |  | x | x |  |  |  |  |  |
| S1 | 35864528 | Activator |  |  | x |  | x |  |  |  |  |
|  | 30500980 | Inhibitor |  |  | x | x |  |  |  |  |  |

^a^PMID: PubMed identifier.

**Supplemental table 2: List of positional weight matrix (PWM) identifiers used for *in silico* analyses.**

| PWM ID | Name | Species | Class | Family |
| --- | --- | --- | --- | --- |
| MA0030.1 | FOXF2 | Homo sapiens | Fork head/winged helix factors | FOX |
| MA0031.1 | FOXD1 | Homo sapiens | Fork head/winged helix factors | FOX |
| MA0032.1 | FOXC1 | Homo sapiens | Fork head/winged helix factors | FOX |
| MA0033.1 | FOXL1 | Homo sapiens | Fork head/winged helix factors | FOX |
| MA0041.2 | FOXD3 | Homo sapiens | Fork head/winged helix factors | FOX |
| MA0042.1 | FOXI1 | Homo sapiens | Fork head/winged helix factors | FOX |
| MA0047.3 | FOXA2 | Homo sapiens | Fork head/winged helix factors | FOX |
| MA0148.1 | FOXA1 | Homo sapiens | Fork head/winged helix factors | FOX |
| MA0157.2 | FOXO3 | Homo sapiens | Fork head/winged helix factors | FOX |
| MA0479.1 | FOXH1 | Homo sapiens | Fork head/winged helix factors | FOX |
| MA0481.1 | FOXP1 | Homo sapiens | Fork head/winged helix factors | FOX |
| MA0593.1 | FOXP2 | Homo sapiens | Fork head/winged helix factors | FOX |
| MA0613.1 | FOXG1 | Homo sapiens | Fork head/winged helix factors | FOX |
| MA0845.1 | FOXB1 | Homo sapiens | Fork head/winged helix factors | FOX |
| MA0846.1 | FOXC2 | Homo sapiens | Fork head/winged helix factors | FOX |
| MA0847.1 | FOXD2 | Homo sapiens | Fork head/winged helix factors | FOX |
| MA0848.1 | FOXO4 | Homo sapiens | Fork head/winged helix factors | FOX |
| MA0849.1 | FOXO6 | Homo sapiens | Fork head/winged helix factors | FOX |
| MA0850.1 | FOXP3 | Homo sapiens | Fork head/winged helix factors | FOX |
| MA0852.2 | FOXK1 | Homo sapiens | Fork head/winged helix factors | FOX |
| MA1103.1 | FOXK2 | Homo sapiens | Fork head/winged helix factors | FOX |
| MA1487.1 | FOXE1 | Homo sapiens | Fork head/winged helix factors | FOX |
| MA1489.1 | FOXN3 | Homo sapiens | Fork head/winged helix factors | FOX |
| MA1683.1 | FOXA3 | Homo sapiens | Fork head/winged helix factors | FOX |
| UN0123.1 | FOXR2 | Homo sapiens | Fork head/winged helix factors | FOX |
| UN0537.1 | FOXP4 | Homo sapiens | Fork head/winged helix factors | FOX |
| UN0538.1 | FOXS1 | Homo sapiens | Fork head/winged helix factors | FOX |
| MA0523.1 | TCF7L2 | Homo sapiens | High-mobility group (HMG) domain factors | TCF-7-related factors |
| MA0768.1 | LEF1 | Homo sapiens | High-mobility group (HMG) domain factors | TCF-7-related factors |
| MA0769.2 | TCF7 | Homo sapiens | High-mobility group (HMG) domain factors | TCF-7-related factors |
| MA1421.1 | TCF7L1 | Homo sapiens | High-mobility group (HMG) domain factors | TCF-7-related factors |

**References**

1. Li, X., Wang, W., Wang, J., Malovannaya, A., Xi, Y., Li, W., Guerra, R., Hawke, D. H., Qin, J., and Chen, J. (2015) Proteomic analyses reveal distinct chromatin-associated and soluble transcription factor complexes. *Mol Syst Biol* **11**, 775

2. Tsitsiridis, G., Steinkamp, R., Giurgiu, M., Brauner, B., Fobo, G., Frishman, G., Montrone, C., and Ruepp, A. (2023) CORUM: the comprehensive resource of mammalian protein complexes-2022. *Nucleic Acids Res* **51**, D539-D545

3. Moparthi, L., Pizzolato, G., and Koch, S. (2019) Wnt activator FOXB2 drives the neuroendocrine differentiation of prostate cancer. *Proc Natl Acad Sci U S A* **116**, 22189-22195

4. Moreira, S., Seo, C., Gordon, V., Xing, S., Wu, R., Polena, E., Fung, V., Ng, D., Wong, C. J., Larsen, B., Raught, B., Gingras, A.-C., Lu, Y., and Doble, B. W. (2018) Endogenous BioID elucidates TCF7L1 interactome modulation upon GSK-3 inhibition in mouse ESCs. *bioRxiv*, 431023

5. Pizzolato, G., Moparthi, L., Soderholm, S., Cantu, C., and Koch, S. (2022) The oncogenic transcription factor FOXQ1 is a differential regulator of Wnt target genes. *J Cell Sci* **135**, jcs.260082
